# Conformational ensembles in *Klebsiella pneumoniae* FimH impact uropathogenesis

**DOI:** 10.1073/pnas.2409655121

**Published:** 2024-09-17

**Authors:** Edward D. B. Lopatto, Jerome S. Pinkner, Denise A. Sanick, Robert F. Potter, Lily X. Liu, Jesús Bazán Villicaña, Kevin O. Tamadonfar, Yijun Ye, Maxwell I. Zimmerman, Nathaniel C. Gualberto, Karen W. Dodson, James W. Janetka, David A. Hunstad, Scott J. Hultgren

**Affiliations:** ^a^Department of Molecular Microbiology, Washington University School of Medicine, St. Louis, MO 63110; ^b^Center for Women’s Infectious Disease Research, Washington University School of Medicine, St. Louis, MO 63110; ^c^Department of Pediatrics, Washington University School of Medicine, St. Louis, MO 63110; ^d^Department of Biochemistry and Molecular Biophysics, Washington University School of Medicine, St. Louis, MO 63110

**Keywords:** UTI, mechanism of bacterial colonization, bacterial pathogenesis

## Abstract

*Klebsiella pneumoniae* is an important pathogen causing difficult-to-treat urinary tract infections (UTIs). Over 1.5 million women per year suffer from recurrent UTI, reducing quality of life and causing substantial morbidity and mortality, especially in the hospital setting. Uropathogenic *E. coli* (UPEC) is the most prevalent cause of UTI. Like UPEC, *K. pneumoniae* relies on type 1 pili, tipped with the mannose-binding adhesin FimH, to cause cystitis. However, *K. pneumoniae* FimH is a poor binder of mannose, despite a mannose-binding pocket identical to UPEC FimH. FimH is composed of two domains that are in an equilibrium between tense (low-affinity) and relaxed (high-affinity) conformations. Substantial interdomain interactions in the tense conformation yield a low-affinity, deformed mannose-binding pocket, while domain–domain interactions are broken in the relaxed state, resulting in a high-affinity binding pocket. Using crystallography, we identified the structural basis by which domain–domain interactions direct the conformational equilibrium of *K. pneumoniae* FimH, which is strongly shifted toward the low-affinity tense state. Removal of the pilin domain restores mannose binding to the lectin domain, thus showing that poor mannose binding by *K. pneumoniae* FimH is not an inherent feature of the mannose-binding pocket. Phylogenetic analyses of *K. pneumoniae* genomes found that FimH sequences are highly conserved. However, we surveyed a collection of *K. pneumoniae* isolates from patients with long-term indwelling catheters and identified isolates that possessed relaxed higher-binding FimH variants, which increased *K. pneumoniae* fitness in bladder infection models, suggesting that long-term residence within the urinary tract may select for higher-binding FimH variants.

## Significance

*Klebsiella pneumoniae* is recognized by the CDC as a pathogen of urgent concern, due to the increase of multidrug-resistant strains. *K. pneumoniae* use type 1 pili tipped with the two-domain FimH adhesin to cause urinary tract infections (UTIs). FimH interdomain interactions result in a conformational equilibrium between low-affinity and high-affinity forms. We found that the *K. pneumoniae* FimH conformational equilibrium is skewed toward a low-affinity state. Analysis of *K. pneumoniae* isolates from catheterized patients revealed carriage of variant residues that shift FimH into a more high-affinity state, favoring persistence in the urinary tract. These results provide evidence for the necessity of a fine-tuned FimH conformational equilibrium in uropathogenesis and implicate FimH as a therapeutic target to neutralize *K. pneumoniae* UTIs.

Urinary tract infections (UTIs) afflict over 50% of women at least once within their lifetime and cause significant morbidity and economic costs (1). In addition, in the United States, over 25% of adult sepsis cases originate from UTIs (2). *Klebsiella pneumoniae* is the second-most prevalent causative agent of UTI, after uropathogenic *Escherichia coli* (UPEC), and has increased relative prevalence in complicated UTIs, such as those in chronically ill, diabetic, and catheterized patients, who have increased risk for morbidity (3, 4). Both UPEC and *K. pneumoniae* are becoming increasingly antibiotic-resistant, necessitating a better understanding of *K. pneumoniae* pathogenesis in order to target virulence factors with antibiotic-sparing therapeutics. For example, candidates targeting the type 1 pilus adhesin FimH, such as mannosides and vaccines, have shown promising results in mouse models and early-stage human clinical trials to treat and prevent UPEC UTI (5–7).

Like UPEC, *K. pneumoniae* are reliant on FimH-tipped type 1 pili to bind to and invade bladder epithelial cells, a process that is critical for infection of the urinary tract (8). The type 1 pilus is a prototypical example of a chaperone-usher pathway (CUP) pilus. All CUP gene clusters encode a periplasmic chaperone and an outer membrane usher that function as a molecular machine to assemble pili. Pilin subunits adopt incomplete immunoglobulin (Ig) folds missing the C-terminal beta strand and are unable to properly fold when expressed alone, leading to their degradation (9). The chaperone is composed of two complete Ig domains arranged in a boomerang shape. Chaperones facilitate donor strand complementation in which the edge beta strand of the N-terminal domain of the chaperone transiently completes the Ig fold of each pilin to enable folding of the subunit on the chaperone template (10–12). Chaperone–subunit complexes are targeted to the outer membrane usher, whose periplasmic domains catalyze donor strand exchange (DSE) (13–15). In DSE, all pilin subunits (excluding the tip adhesin) have an N-terminal extension (Nte) that completes the Ig fold of the preceding pilin, while zippering off the chaperone (16). The two-domain FimH adhesin is required to activate the usher, thereby forcing localization of FimH at the tip of the type 1 pilus via DSE with the adaptor FimG, which links FimH to the pilus (17, 18). The N-terminal mannose-binding lectin domain of FimH (FimH_LD_) mediates binding to mannose-decorated uroplakins on the surface of bladder epithelial cells and desmoglein-2 in renal epithelial cells, interactions that are essential for disease (19–21) and can trigger invasion of both UPEC and *K. pneumoniae* into bladder epithelial cells where they subsequently replicate and form clonal intracellular bacterial communities (IBCs) (22–24). IBCs are biofilm-like structures that protect bacteria from immune cells and antibiotics, which, if not effectively treated, can lead to unchecked chronic UTI (22, 25, 26).

While the FimH_LD_ binding pocket mediates direct stereospecific recognition of mannose, the interaction of the lectin domain with the pilin domain greatly influences the conformation of the mannose-binding pocket of FimH. UPEC FimH has been shown to exist in a dynamic conformational equilibrium between a low-affinity tense state and a high-affinity relaxed state (27). In the low-affinity tense state, the FimH lectin domain has a shallow mannose-binding pocket and is dynamically constrained due to interactions with the pilin domain (27). In contrast, in the high-affinity relaxed state, the lectin domain forms a deep, high-affinity mannose-binding pocket, and the orientation of the two domains relative to one another is highly dynamic, sampling various bends and twists (27). The transition in the FimH conformational landscape between the tense and relaxed conformation states is influenced by interactions with its mannose ligand and allosteric interactions between the lectin and pilin domains, which are in turn influenced by chaperone binding, shear force, and natural amino acid variation. First, in an induced-fit mechanism, mannose binding can trigger structural perturbations that result in conversion to the high-affinity relaxed state (27, 28). Second, when in complex with the FimC chaperone (FimCH) or in the form of a truncated lectin domain only, the binding pocket of FimH predominantly adopts a high-affinity conformation due to steric separation of the two domains or absence of the pilin domain, respectively (29, 30). Third, in a catch-bond mechanism, shear force on the bound adhesin can physically separate and interrupt the lectin and pilin interactions to convert to the relaxed state (28, 31). Finally, in UPEC isolates, there are positively selected residues outside of the FimH binding pocket that control the native conformational equilibrium by influencing lectin and pilin domain interactions (27, 32, 33). These amino acid changes that alter the conformational equilibrium have direct impacts on virulence. In the UPEC strain UTI89, FimH variant A62S shifts the equilibrium toward the low-affinity tense state, while the variant A27V/V163A shifts the equilibrium toward the high-affinity relaxed state (27, 33). Importantly, both the tense and relaxed-shifted FimH alleles are attenuated in acute and chronic UTI models, indicating that the conformational equilibrium and the ability to shift between states is important for UPEC pathogenesis in uncomplicated UTI (27, 32, 33). However, in a mouse model of catheter-associated UTI (CAUTI), in which FimH is also required for catheter colonization, we found that the relaxed-shifted UPEC FimH variant colonized the catheter implant equally well as the wild type (WT) strain, suggesting that conformational equilibrium is under different selection pressures in the context of an indwelling catheter, compared with a naïve bladder (27).

The model *K. pneumoniae* cystitis isolate TOP52 encodes *fimH* having 86% amino acid identity with the well-studied FimH from UTI89, including identical mannose-binding pocket residues; deletion of *fimH* results in attenuated TOP52 bladder infection (8). However, the TOP52 FimH differs in mannose-binding ability from that of UTI89. Unlike UTI89, TOP52 FimH-dependent biofilms are inhibited by heptyl mannose but not methyl mannose, indicating a lower affinity of TOP52 FimH toward methyl mannose moieties (8). Further, TOP52, like most *K. pneumoniae* strains, displays no detectable hemagglutination (HA) of guinea pig erythrocytes in a common assay for measuring UPEC type 1 pili function (8). This lack of HA titer is especially striking considering that even tense-shifted FimH mutants of UTI89 still exhibit a low mannose-sensitive HA titer (32). Overexpression of TOP52 type 1 pili does not restore HA titer suggesting that differences in expression are not responsible for the functional differences (8). Complementing TOP52 Δ*fimH* with UTI89 *fimH* yields a moderate HA titer, suggesting that the most likely explanation is that *K. pneumoniae* FimH adopts a low-affinity mannose-binding phenotype (8).

In this study, we sought to resolve the binding differences between the type 1 pili of *K. pneumoniae* and *E. coli.* We demonstrate that the amino acid sequence of FimH is highly conserved among *K. pneumoniae* strains. We show that the *K. pneumoniae* FimH_LD_ isolated away from the pilin domain primarily adopts a high-affinity mannose-binding conformation, supporting that interactions between the lectin and pilin domains negatively impact the mannose-binding pocket. Further, we find the full-length two-domain *K. pneumoniae* FimH at the tip of the pilus is strongly shifted toward a low-affinity tense conformation compared to UPEC FimH. We sequenced *fimH* from *K. pneumoniae* clinical isolates and identified naturally occurring, higher-binding *K. pneumoniae* FimH variants that exhibited enhanced fitness in murine models of UTI. These results show the importance of the FimH conformational equilibrium in UTIs and provide an explanation for the lower relative prevalence of *K. pneumoniae* compared to UPEC in uncomplicated UTI.

## Results

### Majority *of K. pneumoniae* Strains Carry an Identical FimH Sequence.

**Fig. 1.**
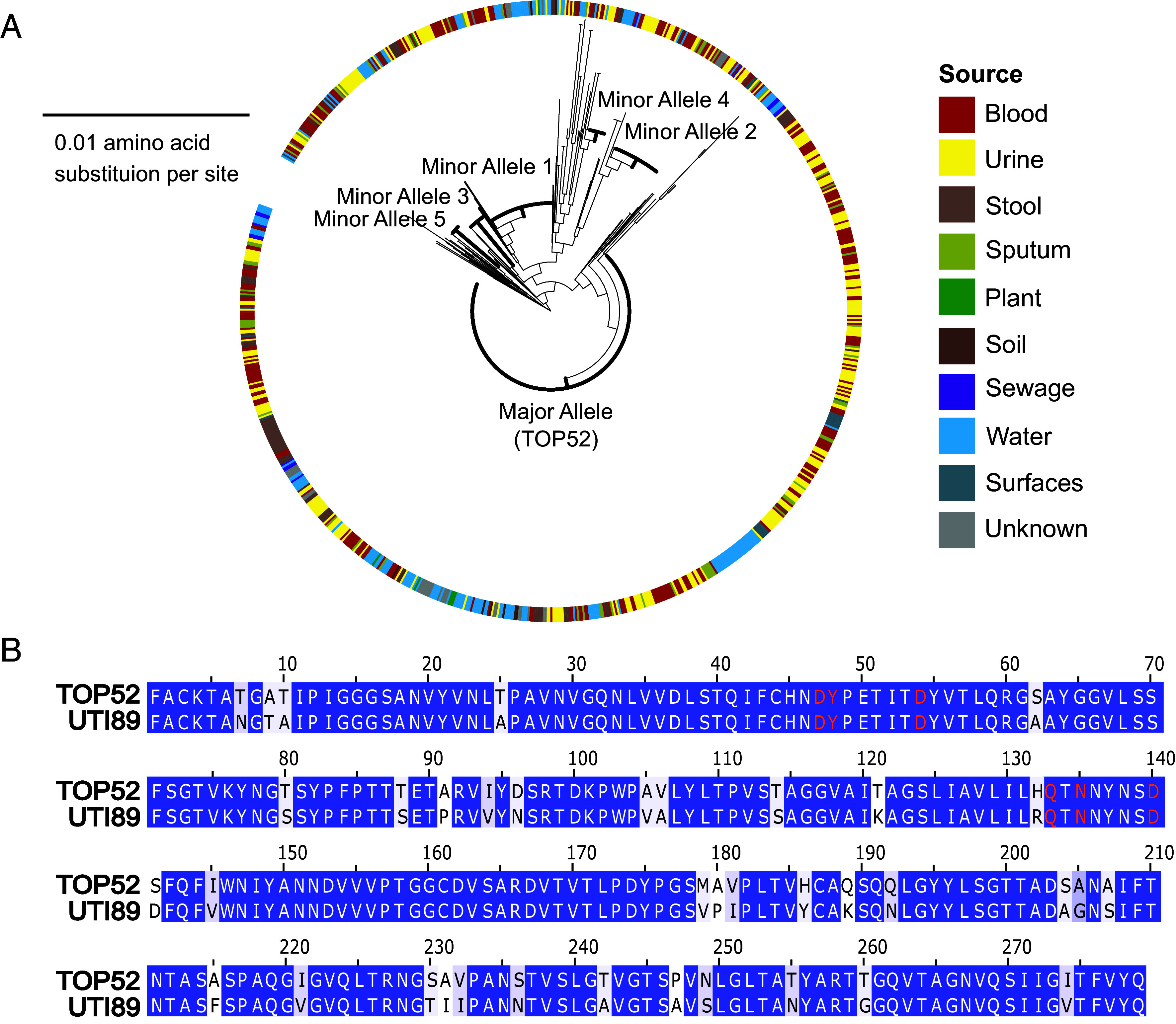
*K. pneumoniae* FimH is highly invariant and conserved among diverse strains. (*A*) Phylogeny of *K. pneumoniae* FimH amino acid sequences (n = 977 strains). Source of strain isolation is annotated by color on outside ring. Minor alleles and strain diversity are shown in *SI Appendix*, Fig. S1. (*B*) Amino acid alignment of *K. pneumoniae* TOP52 FimH and UPEC UTI89 FimH. Blue highlighted residues are identical between sequences, light blue highlighted residues differ but share biochemical properties, and white background denotes different residue with different charge or hydrophobicity. Residues involved in binding to mannose are in orange.

Previous reports have found UPEC FimH and *K. pneumoniae* FimH amino acid sequences are highly conserved with variation in only a few residues (32, 34); however, these analyses were performed on only small sample sizes of clinical isolates. We sought to quantify the diversity of FimH alleles in *K. pneumoniae* utilizing a larger array of publicly available whole-genome sequence data of isolates from diverse sources. Querying *fimH* sequences from 977 *K. pneumoniae* genomes curated by Institut Pasteur showed that all genomes contained a *fimH* gene and over 70% (683/977) of *K. pneumoniae* had an identical FimH amino acid sequence to TOP52, despite a highly diverse set of core genes, positing that this sequence of FimH is important for *K. pneumoniae* fitness (Fig. 1*A* and *SI Appendix*, Fig. S1). The consensus *K. pneumoniae* FimH sequence differs from UTI89 FimH at 38 residues across the 279-residue FimH protein, and all differences are located outside of the mannose-binding pocket (Fig. 1B). The residues involved in binding of mannose are identical to UPEC FimH and identical among the *K. pneumoniae* sequences. We found no association between *K. pneumoniae* isolate source and FimH amino acid sequence. We searched across *E. coli* sequences for the major and five most common minor *K. pneumoniae* FimH alleles using NCBI protein BLAST; we found only one exact match for the major allele and no matches for the minor alleles, suggesting that the *K. pneumoniae* FimH sequence is species specific. Overall, these findings suggest that FimH is generally important for *K. pneumoniae* across niches, and the phenotypes of TOP52 FimH are likely generalizable to most *K. pneumoniae* strains.

### Binding Loop Dynamics in the FimH Lectin Domain Affect Mannose Binding.

**Fig. 2.**
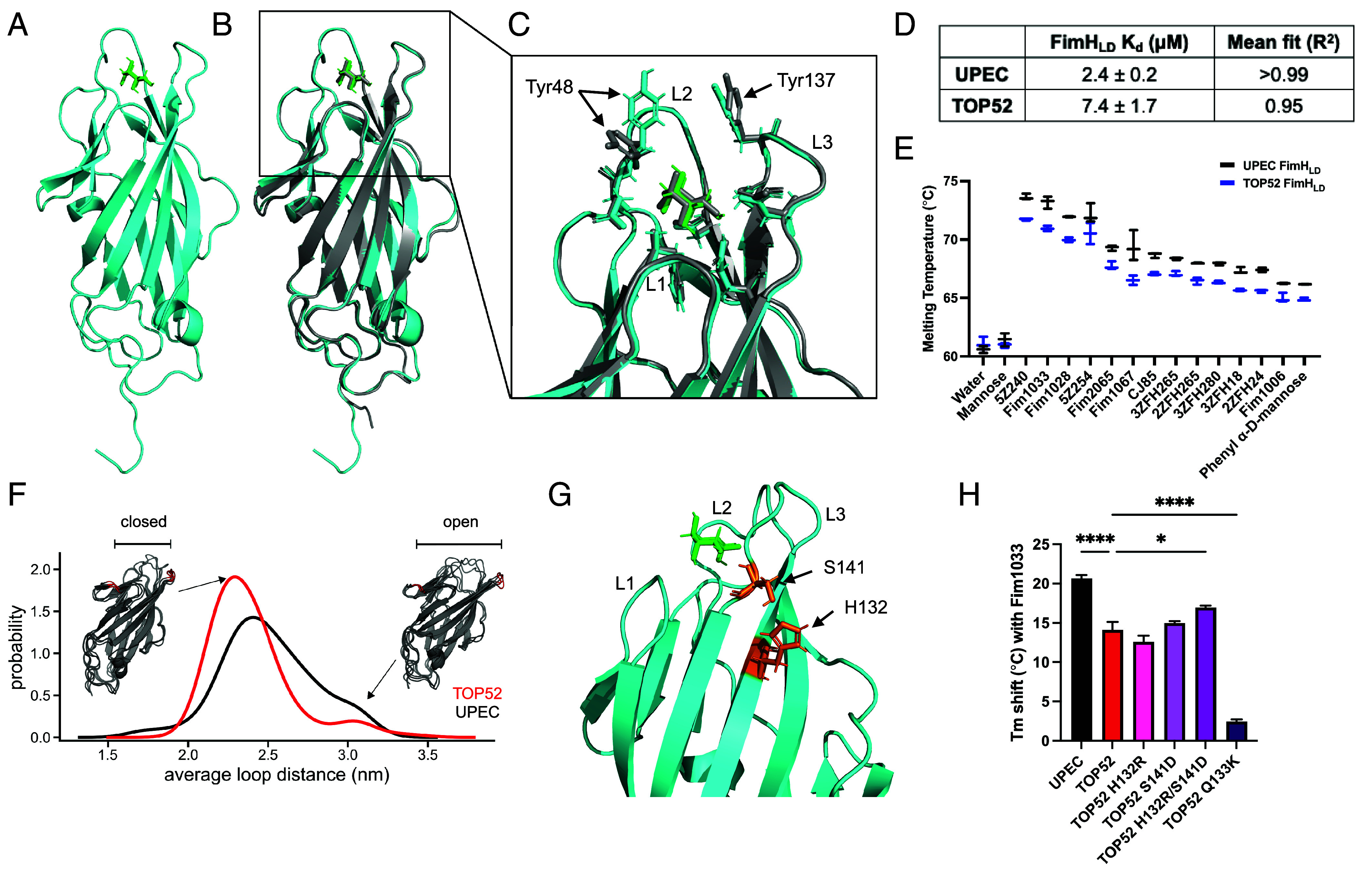
*K. pneumoniae* FimH_LD_ displays high-affinity binding to mannose with altered binding loop 3 dynamics. (*A*) Crystal structure of *K. pneumoniae* TOP52 FimH_LD_ (cyan) bound to D-mannose (green). (*B*) Alignment of UPEC FimH_LD_ bound to D-mannose (gray; PDB 1KLF FimH_LD_) to the TOP52 FimH_LD_ structure (RMSD 0.420). (*C*) Comparison of binding pocket between UPEC and *K. pneumoniae* FimH_LD_. Binding loops are denoted L1, L2, and L3. (*D*) Observed BLI K_d_ of FimH_LD_ to BSA-mannose. (*E*) DSF binding of UPEC (black) and *K. pneumoniae* FimH_LD_ (blue) to an array of chemically diverse mannosides (n = 3). (*F*) FAST simulation population distribution of pocket distance from base of L1 to L3 for UPEC FimH_LD_ (gray) and *K. pneumoniae* FimH_LD_ (red). (*G*) Residue differences at 132 and 141 at the base of binding loop 3 on *K. pneumoniae* FimH_LD_ from the residues at these positions in UPEC FimH_LD_ (PDB 1KLF FimH_LD_). (*H*) DSF binding for TOP52 FimH_LD_ binding loop three mutants (n = 3). Error bars represent SEM. ANOVA, *P ≤ 0.05, **P ≤ 0.01, ***P ≤ 0.001, ****P ≤ 0.0001.

We investigated the structure of the mannose-binding FimH lectin domain truncate (FimH_LD_) of *K. pneumoniae*, which is separated from any allosteric effects of the FimH pilin domain. We solved the X-ray crystal structure of TOP52 FimH_LD_ bound to d-mannose to 1.34 Å (Fig. 2*A*). A comparison of the structures of UPEC and *K. pneumoniae* FimH_LDs_ revealed that both FimHs bind d-mannose in a similar fashion with small differences in the orientation of Y48 and Y137, located on binding loops 2 and 3 respectively, which make up a “tyrosine gate” and are involved in binding of mannose ligands (19, 35) (Fig. 2 *B* and *C*). In *K. pneumoniae* FimH_LD_, Y48 and Y137 are ~4.2 Å closer to each other compared to UPEC FimH_LD_. In agreement with the structural similarity, measurements of binding to BSA-mannose by biolayer interferometry showed that *K. pneumoniae* FimH_LD_ bound to mannose with high affinity (K_d_ = 7.4 μM) although slightly lower than UPEC FimH_LD_ (K_d_ = 2.4 μM; Fig. 2*D*). By differential scanning fluorimetry (DSF), a panel of structurally different high-affinity mannosides also showed modestly higher binding to UPEC FimH_LD_ than to *K. pneumoniae* FimH_LD_ (Fig. 2*E* and *SI Appendix*, Table S1).

The differing mannose affinities between *K. pneumoniae* FimH_LD_ and UPEC FimH_LD_, combined with the different orientations of the tyrosine gate, prompted us to explore the possibility that *K. pneumoniae* FimH_LD_ may have altered binding loop dynamics. When the mannose was computationally removed from the FimH_LD_ structures and molecular dynamics of the apo lectin domains were analyzed using a fluctuation amplification of specific traits (FAST) simulation, we found only one major difference in the equilibrium of *K. pneumoniae* FimH_LD_ compared to UPEC FimH_LD_. The simulation suggested that loop 3 had increased flexibility in UPEC FimH_LD_ compared to *K. pneumoniae* FimH_LD_, as UPEC FimH_LD_ had an increased number of states with loop 3 in an open position (Fig. 2*F*). Close examination of the crystal structure showed two residue interaction differences at positions 132 and 141 from UPEC at the base of binding loop 3, between the *K. pneumoniae* residues (H132 and S141) compared to the analogous interaction in UPEC (R132 and D141; Fig. 2*G*). To test whether interactions at positions 132 and 141 contributed to differences in mannose binding, we mutated the *K. pneumoniae* FimH_LD_ residues singly and in combination toward the residues found in UPEC FimH and then assayed mannose binding of these variants by DSF. We found that only the *K. pneumoniae* FimH_LD_ H132R/S141D double mutant significantly enhanced binding, reflected by an increase in melting temperature by 2.8 °C for the FimH_LD_ in complex with the high-affinity mannoside Fim1033 (36). These results suggest that the H132/S141 residues in *K. pneumoniae* FimH_LD_ result in a less flexible binding loop 3 that reduces mannose-binding affinity. Both H132 and S141 were conserved among all *K. pneumoniae* genomes in our analysis (Fig. 1*A* and *SI Appendix*, Fig. S1). Together, these results suggest that the *K. pneumoniae* FimH_LD_ is capable of binding to mannose with high affinity, with differences in absolute affinity relative to UPEC FimH_LD_ being partially explained by binding loop 3 dynamics.

### *K. pneumoniae* Isolated from Catheter Infections Contain High-Affinity FimH Variants.

**Fig. 3.**
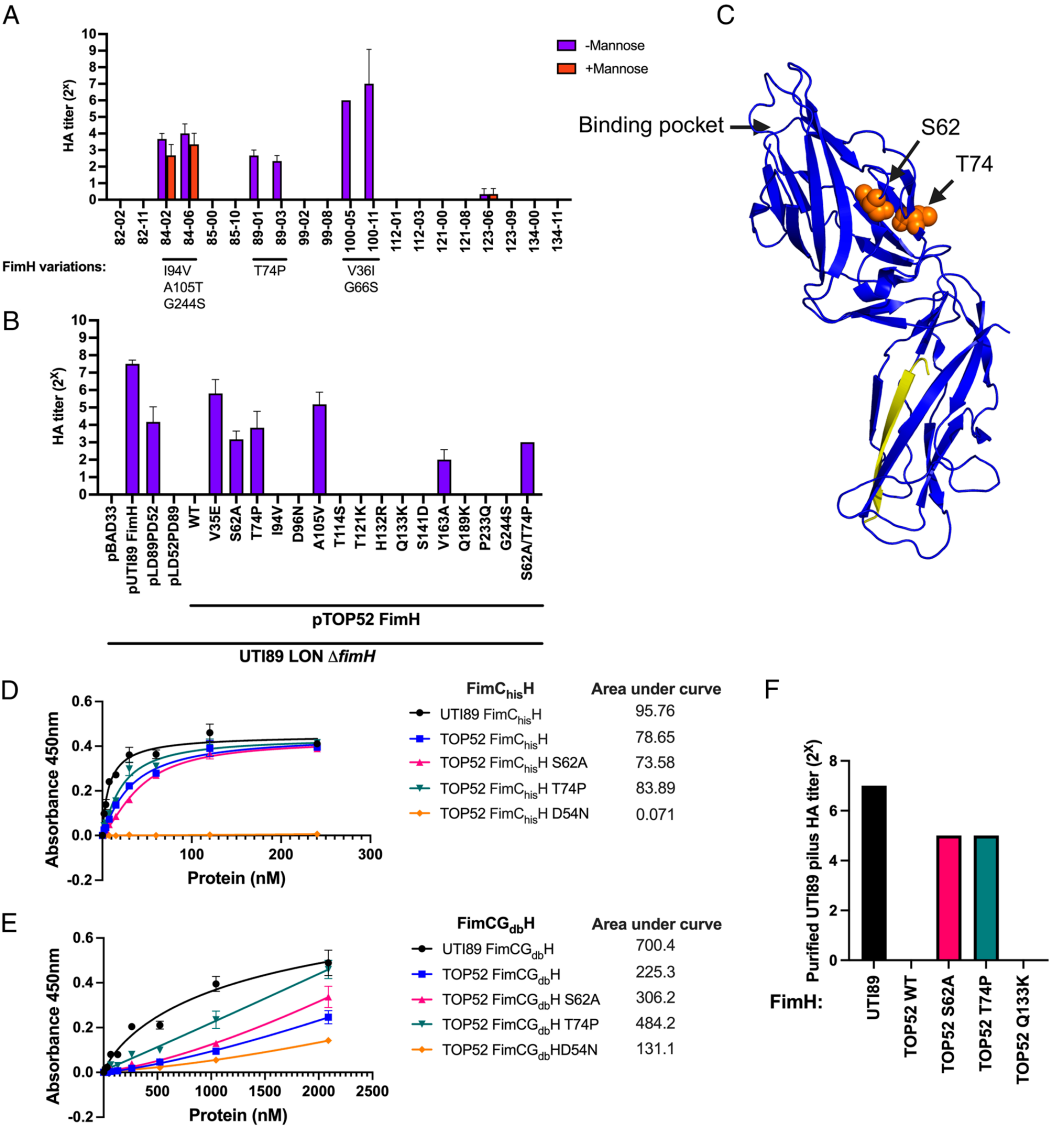
*K. pneumoniae* TOP52 FimH is allosterically shifted toward a tense low-affinity conformation and variants can shift FimH toward a higher-affinity conformation. (*A*) HA titers of clinical CAUTI *K. pneumoniae* isolates with FimH variants (no listed variant means the strain has the same sequence as TOP52; n = 2 to 3). (*B*) HA titer FimH variants expressed from plasmids in UTI89 LON *ΔfimH* (n = 3). (*C*) Positions of S62A and T74P on the structure of UPEC FimH in tense conformation (PDB 5JQI). (*D* and *E*). ELISA binding curves of FimC_his_H variants (*D*) and FimCG_db_H variants (*E*). Area under the curve measurements are displayed next to each respective graph. (*F*) HA titers of purified UPEC type 1 pili tipped with FimH variants. All HA titers were completely inhibited by the addition of 100 mM methyl α-D-mannopyranoside. Error bars represent SEM.

We previously found that patients with long-term indwelling urinary catheters can be continuously colonized with *K. pneumoniae* species (37). To assess whether these isolates contain FimH, we selected 10 patients colonized with *K. pneumoniae* and isolated the first and last strains from the catheters of those patients; resulting in 20 *K. pneumoniae* isolates (*SI Appendix*, Table S2*)*. The time between the first and last samples for an individual patient ranged from 59 to 637 d, with an average catheter indwelling time of 30 d between samples (37), representing a significant period of ongoing infection within the patients (*SI Appendix*, Table S2). During these long-term infection periods, patients often underwent multiple courses of antibiotics, some of which did not clear the infection (*SI Appendix,* Table S2). PCR amplification showed that all 20 *K. pneumoniae* urinary isolates encoded a *fimH* gene. Further, the *fimH* sequences in the strains from the first and last collection periods were identical for every patient. Most of the isolates encoded the same FimH amino acid sequence as TOP52, but six isolates harbored variations from TOP52 (Fig. 3*A*). Isolates from patient 89 carried a single amino acid variant from TOP52 FimH (T74P), while the others had two or more variations (I94V/A105T/G244S and V36I/G66S) from the TOP52 FimH sequence. We found that seven isolates from four different patients (patients 84, 89, 100, 123) were able to agglutinate guinea pig erythrocytes, with four isolates producing mannose-inhibitable HA, indicating involvement of type 1 pili (Fig. 3*A*); these four isolates all had amino acid variations from TOP52 FimH. These results argue that clinical *K. pneumoniae* isolates can encode natural FimH variants outside of the binding pocket that can enhance mannose binding and are present at multiple longitudinal collections within the same patient.

To specifically test the effects of FimH variation in an otherwise isogenic background, FimH variants of interest were encoded in TOP52 *fimH* and used to complement a UTI89 type 1 operon overexpression strain, in which the left inverted repeat of the *fimS* promoter switch is genetically engineered to force type 1 pilus expression (“locked on” [LON]), with a *fimH* deletion. In this assay, expressing WT UTI89 *fimH* restored the HA titer to 8. In contrast, expressing WT TOP52 *fimH* was unable to complement the HA titer (Fig. 3*B*). Expressing a *fimH* chimeric allele, consisting of the TOP52 FimH_LD_ with the UTI89 pilin domain, did not produce an HA titer. However, expressing a chimeric allele composed of the UTI89 FimH_LD_ and the TOP52 pilin domain weakly restored HA activity. While not all TOP52 *fimH* variants produced an HA titer, variants V35E, S62A, T74P, A105V in the lectin domain and variant V163A in the pilin domain yielded moderate hemagglutination, suggesting that variants outside of the binding pocket can transition FimH to a higher-affinity conformation. Complementation with TOP52 *fimH*, both UTI89 and TOP52 *fimH* chimeras, or TOP52 *fimH_S62A_* and *fimH_T74P_* variants resulted in fewer pili than WT UTI89 *fimH*, indicating a defect in the ability of TOP52 FimH to initiate usher activation and thus piliation (*SI Appendix*, Fig. S2*A*). This finding indicates that residue changes in FimH may adversely affect pilus assembly initiation and that alleles that fail to complement HA ability may reflect either assembly or binding defects. Interestingly, TOP52 FimH variants that did restore hemagglutination encoded residues previously implicated in influencing the conformational equilibrium of UPEC FimH between high and low binding affinity, such as V35E and S62A (27, 38). These findings suggest that while most *K. pneumoniae* encode a FimH variant that is conformationally shifted to a low binding state, some clinical isolates carry mutations that result in a higher-affinity conformation.

### FimH Mutations Allosterically Increase *K. pneumoniae* FimH Binding Affinity.

From knowledge of the UPEC FimH equilibrium, our analysis of 977 *K. pneumoniae fimH* sequences in the database, and our CAUTI isolate analysis, we selected two *K. pneumoniae* FimH variants (S62A and T74P) for further study (Fig. 3C). Both FimH variants produced an HA titer when expressed in UTI89 LON Δ*fimH* and were present in our analysis among the 977 strains in the genome database. In UTI89, we found that FimH A62S shifts the conformational equilibrium strongly toward a low-affinity tense state (27, 33); we therefore hypothesized that an S62A mutation in *K. pneumoniae* FimH would yield a more high-affinity FimH conformation. The FimH T74P variant also appeared in a CAUTI isolate that produced a mannose-sensitive HA titer, suggesting that this variant results in a relaxed-shifted *K. pneumoniae* FimH conformational equilibrium. As null-binding controls, we also investigated binding-pocket mutants (D54N and Q133K) that abrogate FimH binding to mannose (19).

To test the conformational effects on the relative binding of purified FimH protein variants, we assessed binding of purified TOP52 FimH variants (WT, S62A, T74P, and D54N) to bovine submaxillary mucin (BSM), a direct measure of binding to a glycoprotein separate from the differential effects of FimH variants on pilus initiation and incorporation, in the context of i) purified FimC_his_H complexes, in which all variants should bind mannose with high affinity, as the FimC chaperone binding FimH prevents allosteric interactions between the FimH pilin and lectin domains, favoring the high-affinity relaxed conformation; and ii) in the context of a “tip-like complex” in which FimH has undergone DSE with FimG (as it does at the tips of type 1 pili) and is thus free to adopt the low- or high-affinity state. FimC_his_H complexes were purified by cobalt affinity; TOP52 FimC_his_H_D54N_ was chosen as the nonbinding control for purified FimH protein studies due to low yields of TOP52 FimC_his_H_Q133K_. To produce purified FimH in a tip-like state in which the FimH lectin and pilin domains can interact, we performed in vitro DSE between FimC_his_H and disulfide-bond crosslinked UTI89 FimC_A106C-his_G_S138C_ (hereafter termed FimCG_db_) to create FimCG_db_H (*SI Appendix*, Fig. S3). Briefly, when FimC_his_H and FimC_his_G are mixed in solution, the Nte of FimG displaces the donated FimC_his_ β-strand on FimH to produce free FimC_his_ and tip-like FimC_his_GH. However, in vitro DSE between FimC_his_H and FimC_his_G produces FimC_his_GH along with by-products of higher-order structures with multiple FimG subunits such as FimC_his_GGH and FimC_his_GGGH (33). Thus, to favor the formation of FimC_his_GH complexes, FimCG_db_ was engineered with disulfide bonding of FimC to FimG. This prevents release of FimC from the FimCG_db_ construct and precludes DSE between FimG with other FimGs, yielding a singular FimCG_db_H DSE product when mixed with FimC_his_H (*SI Appendix*, Fig. S3*C*). UTI89 FimC_his_H bound to BSM with high affinity (area under the curve (AUC) = 95.76; Fig. 3*D*). TOP52 WT FimC_his_H, FimC_his_H_S62A_, and FimC_his_H_T74P_ bound with similar high affinity to BSM (AUCs between 73 and 83). As expected, TOP52 FimC_his_H_D54N_ showed no binding (AUC = 0.07). When the corresponding tip-like UTI89 and TOP52 FimCG_db_H complexes were assayed for binding to BSM, UTI89 FimCG_db_H (AUC = 700.4) bound with much higher affinity than WT TOP52 FimCG_db_H (AUC = 225.3; Fig. 3*E*). TOP52 FimCG_db_H_D54N_ displayed low binding (AUC = 131.1). TOP52 FimCG_db_H_S62A_ (AUC = 306.2) and TOP52 FimCG_db_H_T74P_ (AUC = 484.2) resulted in greatly higher binding than WT TOP52 FimCG_db_H, but still had less adherence than UTI89 FimCG_db_H.

To further probe the binding affinity of tip-like FimH, we purified pili from UTI89 LON Δ*fimH* complemented with TOP52 FimH WT, S62A, T74P, and Q133K variants and measured their ability to agglutinate guinea pig erythrocytes when made polyvalent by the addition of FimA antisera. The amount of FimA was normalized by protein absorbance at 280 nm and visualized by SDS-PAGE (*SI Appendix*, Fig. S2*B*). In this system, pili with UTI89 FimH produced an HA titer of 7, while pili with WT TOP52 FimH unexpectedly lacked an HA titer (Fig. 3*F*). Purified pili tipped with TOP52 FimH_S62A_ and TOP52 FimH_T74P_ produced a moderate HA titer of 5, and pili tipped with TOP52 FimH_Q133K_ produced no HA titer. All HA titers were fully inhibited by methyl α-D-mannopyranoside. When pili were blotted to measure incorporation of FimH, WT TOP52 FimH and its variants were less efficient at being incorporated into the pilus, further indicating an assembly defect and likely indicating this assay is underestimating the binding ability of the TOP52 FimH variants (*SI Appendix*, Fig. S2*B*). All together, these results suggest that WT TOP52 FimH is naturally in a low-affinity mannose-binding state when incorporated into type 1 pili and that S62A and T74P mutations can allosterically shift purified tip-like FimH to a higher-affinity state.

### Type 1 Pili Binding and Expression in *K. pneumoniae* with Allosterically Relaxed-Shifted FimH Variants.

**Fig. 4.**
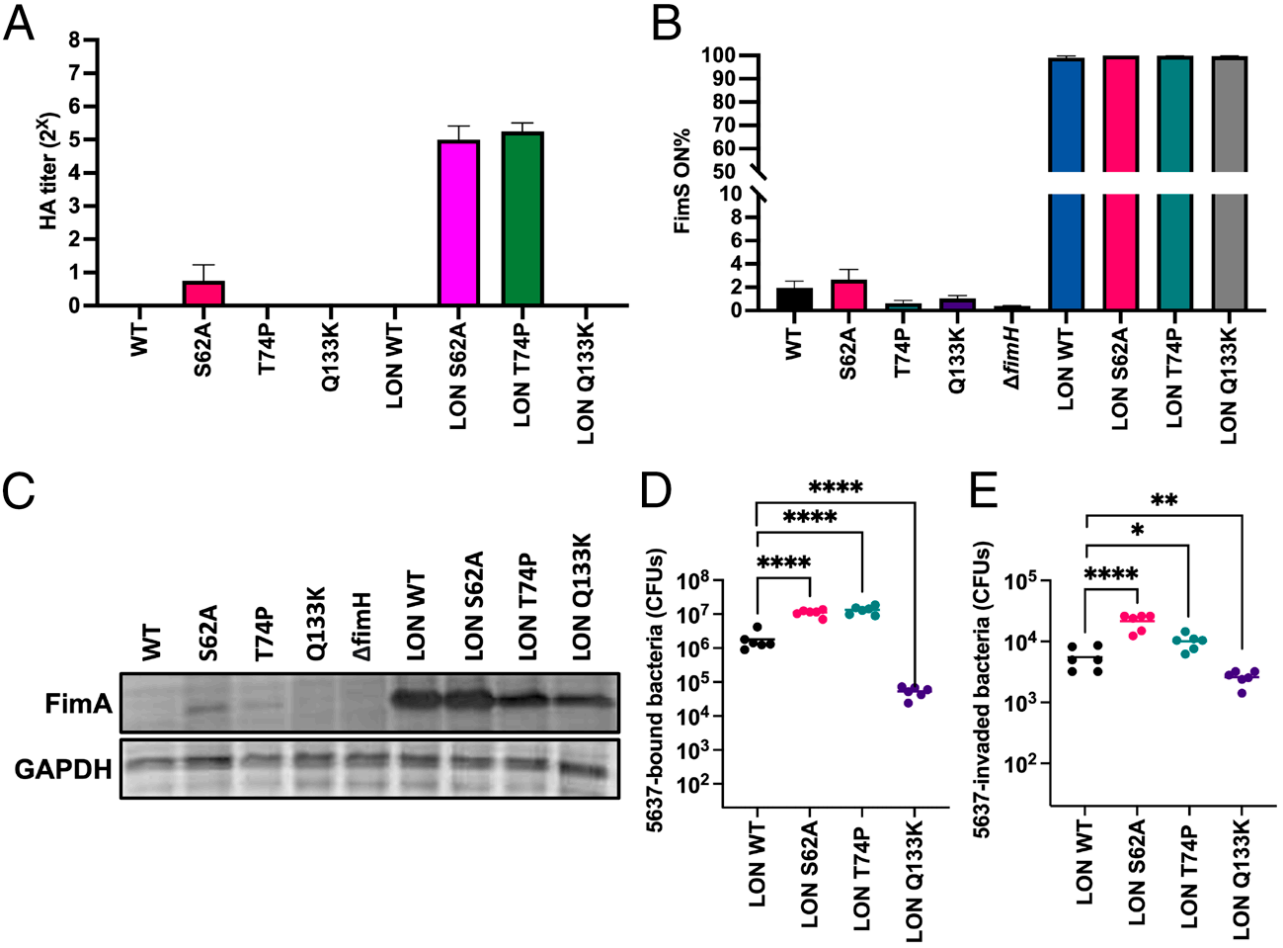
Relax-shifted FimH variants increase *K. pneumoniae* type 1 pilus binding. (*A*) HA titers of chromosomal TOP52 FimH variants. LON indicates FimS promotor locked in on orientation to overexpress type 1 pili. HA titers were completely inhibited by the addition of 100 mM methyl α-D-mannopyranoside (n = 3). (*B*) FimS on percentage (n = 3). (*C*). Western blot of FimA and GAPDH loading control of TOP52 FimH variants. (*D*) 5637-cell attachment and invasion (*E*) of TOP52 LON FimH variants (n = 6). ANOVA, *P ≤ 0.05, **P ≤ 0.01, ***P ≤ 0.001, ****P ≤ 0.0001.

To explore how FimH_S62A_ and FimH_T74P_ alter *K. pneumoniae* type 1 pili–mediated pathogenesis, we chromosomally engineered these mutations and the nonbinding FimH_Q133K_ into TOP52. We then assayed HA titers after growth in static LB broth (type 1 pili–inducing condition for UPEC) (39) and only TOP52 *fimH*_S62A_ observed a minimal HA titer of 1 (Fig. 4*A*). In these growth conditions, the TOP52 invertible *fimS* promoter was almost entirely in the OFF orientation, and low amounts of piliation were detected for all strains. Thus, we engineered a *fimS* locked-on (LON) strain of TOP52. Indeed, *fimS* orientation was nearly 100% ON and we were able to detect significant type 1 pilus expression by western blot in the LON strains (Fig. 4 *B* and *C*). When expressed in the TOP52 LON background, *fimH*_S62A_ and *fimH*_T74P_ mutants produced a moderate HA titer of 5, while WT *fimH* and *fimH*_Q133K_ had HA titers of 0. All HA titers were completely inhibited by the addition of methyl α-d-mannopyranoside. As an additional measure of type 1 pili binding, TOP52 LON *fimH*_S62A_ and TOP52 LON *fimH*_T74P_ demonstrated significantly more binding and invasion of 5637 bladder epithelial cells compared to TOP52 LON WT *fimH*, while TOP52 LON *fimH*_Q133K_ exhibited decreased cell attachment and invasion (Fig. 4 *D* and *E*).

### Relaxed-Shifted *K. pneumoniae FimH* Variants Convey Increased Fitness in the Bladder.

**Fig. 5.**
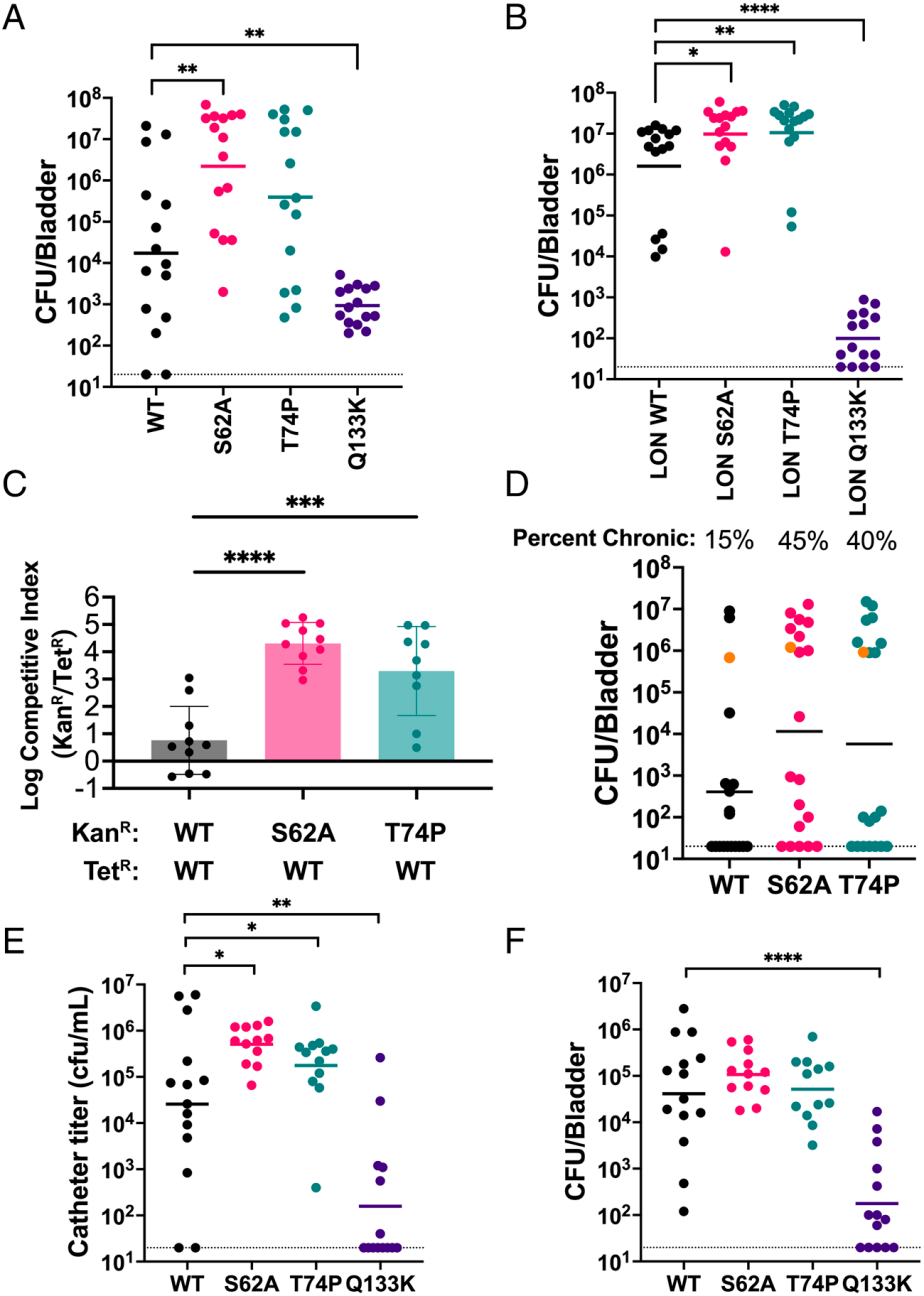
Relaxed-shifted *K. pneumoniae* FimH variants increase bladder and catheter titers relative to TOP52 FimH. (*A* and *B*) C3H/HeN mice infected with 10^9^ CFUs of TOP52 expressing FimH variants and bladder titers enumerated 24 hpi (n = 15 mice total from three independent replicates). (*C*) Log competitive index 2 dpi from a competitive bladder infection in C3H/HeN mice. Error bars represent geometric SD (n = 10 total from two independent replicates). (*D*) Bladder titers and percentage of C3H/HeN mice with chronic bladder infection 28 dpi. Orange dots indicate mice which had high bladder titers at 28 dpi but had at least one urine titer below 10^4^ CFUs during the experiment and therefore did not count as a chronically infected mouse (n = 20 over two independent replicates). (*E* and *F*) C57BL/6 mice were catheterized and infected with 10^7^ CFUs of TOP52 variants. Catheter titers (*E*) and bladder titers (*F*) were enumerated 24 hpi (n = 13 to 14 mice from two independent replicates). Solid lines on all graphs show geometric mean. Dotted lines indicate limit of detection. Mann–Whitney *U* test (except for C, one-way ANOVA), * *P* ≤ 0.05, **P ≤ 0.01, ***P ≤ 0.001, ****P ≤ 0.0001.

In UPEC, the FimH conformational equilibrium is finely tuned and altering the conformational equilibrium toward either a high-affinity relaxed state or a low-affinity tense state attenuates infection in the bladder (32). We hypothesized that the tense-shifted *K. pneumoniae* FimH of TOP52 limits its ability to infect the urinary tract and that variants shifting the FimH conformational equilibrium toward the high-affinity state would enhance the ability of TOP52 to initiate UTI. Thus, we tested the conformationally shifted TOP52 *fimH* variant strains in a murine model of acute UTI in which C3H/HeN mice were transurethrally infected and bladder titers were enumerated 24 h post infection (hpi). In this acute UTI model, WT TOP52 *fimH* displayed highly variable bladder titers, ranging from below the limit of detection to ~10^7^ CFU (Fig. 5*A*). The relaxed-shifted TOP52 *fimH*_S62A_ variant established significantly higher bladder titers compared to WT TOP52 *fimH*, and the relaxed-shifted TOP52 *fimH*_T74P_ variant also trended higher. As expected, TOP52 *fimH*_Q133K_ was significantly attenuated compared to WT TOP52 *fimH*, confirming the pathogenic importance of *K. pneumoniae* type 1 pili in the bladder.

UPEC FimH variants that shift the conformational equilibrium also alter type 1 pilus expression (32). While we did not detect major differences in type 1 pilus expression among FimH variants in TOP52 (owing to low expression of type 1 pili in vitro), we tested TOP52 LON *fimH* strains in the acute UTI model to control for potential variable expression in the urinary tract. TOP52 LON *fimH*_S62A_ and TOP52 LON *fimH*_T74P_ exhibited significantly higher infection titers compared to TOP52 LON WT *fimH* (Fig. 5*B*). Interestingly, TOP52 LON *fimH*_Q133K_ achieved lower colonization compared to the TOP52 *fimH*_Q133K_, suggesting that overexpression of type 1 pili in vivo may influence other *K. pneumoniae* virulence factors.

Next, we performed competitive acute bladder infections to directly compare fitness between FimH alleles using TOP52 strains carrying either a tetracycline resistance gene (tet^R^) or kanamycin resistance gene (kan^R^) inoculated in a 1:1 ratio. No differences were observed when TOP52 WT *fimH* kan^R^ was competed with TOP52 WT *fimH* tet^R^ (Fig. 5*C*). In contrast, TOP52 *fimH*_S62A_ kan^R^ outcompeted TOP52 WT *fimH* tet^R^ by ~4 orders of magnitude. Similarly, TOP52 *fimH*_T74P_ kan^R^ outcompeted TOP52 WT *fimH* tet^R^ by ~3 orders of magnitude. Next, we explored the fitness of these variants in long-term infection. We infected C3H/HeN with the TOP52 FimH variants and tracked the infection over 28 d to determine the percentage of mice that developed chronic cystitis (defined by >10^4^ CFU/mL in the urine and bladder throughout the experiment). While WT TOP52 established chronic infection in 15% of mice, TOP52 *fimH*_S62A_ and *fimH*_T74P_ caused chronic UTI in 45% and 40%, respectively (Fig. 5*D*). Collectively, these results suggest that higher-affinity *K. pneumoniae* FimH variants enable increased fitness in the bladder in both short-term and long-term infection.

### Relaxed-Shifted *K. pneumoniae FimH* Variants Provide Increased Attachment to Catheters.

Clinically, *K. pneumoniae* is a more prevalent etiological agent in complicated UTI (including CAUTI) than in acute uncomplicated UTI (3). We also identified clinical CAUTI *K. pneumoniae* isolates that encoded FimH under positive selection, including the higher-affinity FimH_T74P_ allele. We posited that higher-affinity FimH variants would promote increased colonization of the bladder and catheters in a murine CAUTI model where C57BL/6 mice are implanted with a piece of silicone tubing (40). Compared to WT TOP52 in this model, TOP52 *fimH*_S62A_ and TOP52 *fimH*_T74P_ exhibited significant increases in catheter titers 24 hpi (Fig. 5*E*). However, bladder titers were not significantly different between TOP52 WT and variants (Fig. 5*F*). TOP52 *fimH*_Q133K_ was markedly attenuated in both catheter and bladder titers, demonstrating the pathogenic importance of type 1 pili for *K. pneumoniae* CAUTI. These data suggest that higher-affinity FimH variants, including one variant that was found in a clinical CAUTI isolate, allow *K. pneumoniae* to better attach to catheter material to initiate and perpetuate CAUTI.

## Discussion

*K. pneumoniae* is a major pathogen responsible for bloodstream infections, pneumonia, and UTIs. Surprisingly, despite the ability of this pathogen to colonize multiple infection sites, *K. pneumoniae* is identified as the etiological agent of acute UTI much less often than UPEC (3). Despite the genomic diversity of *K. pneumoniae* compared to UPEC, the *fim* gene clusters encoding type 1 pili, which both pathogens rely on to infect the urinary tract (8), are strikingly conserved among most *K. pneumoniae* strains. *K. pneumoniae* strains have been shown to progress through the same pathogenic cascade as *E. coli,* recapitulating the features of i) FimH-dependent bladder colonization; ii) IBC formation; and iii) filamentation and dispersion of bacteria from the IBC to infect neighboring cells (8, 23). Paradoxically, *K. pneumoniae* FimH is a poor mannose binder despite an identical mannose-binding pocket as UPEC FimH. This study explains the structural basis of this phenotype by elucidating how allosteric effects between the two domains in native *K. pneumoniae* FimH cause the mannose-binding pocket to preferentially adopt a low-affinity binding conformation, thereby partially explaining epidemiologic features of *K. pneumoniae* UTI. However, other virulence factors, such as type 3 pili, are also known to contribute to *K. pneumoniae* UTI virulence (41). Interestingly, we also identified amino acid variations that shift the *K. pneumoniae* FimH conformational equilibrium toward higher affinity and increase the ability of the pathogen to cause bladder infection.

In UPEC, the FimH conformational equilibrium is finely tuned to optimize efficient infection of the urinary tract. FimH alleles that shift this equilibrium toward either lower or higher affinity attenuate infection (32). It is unknown what selective pressures may be acting on *K. pneumoniae* FimH. We show in this study that *K. pneumoniae* FimH most often exists in a tense conformation at the tips of type 1 pili. However, we identified strains with high-affinity FimH variants were readily found in clinical isolates from patients with CAUTI (37), suggesting that this niche may select for variants that will promote persistence in the urinary tract. Specifically, while TOP52 FimH confers low binding to mannose, here we found that TOP52 FimH_T74P_, a variant present in a clinical sample of *K. pneumoniae* from a long-term catheterized patient, was a higher-affinity variant. Thus, FimH_T74P_ represents a naturally occurring, previously uncharacterized allele that shifts the FimH domain equilibrium toward the relaxed conformation, minimizing allosteric interaction of the two domains and enabling high affinity of mannose-binding pocket. The biochemical mechanism of this allosteric shift will need to be further elucidated, as residue 74 does not have major solvent-accessible surface area changes between the tense and relaxed FimH structures, as found in other residue positions influencing FimH conformational equilibrium (38), and the residue change to proline would create new physical constraints on the peptide backbone.

In addition to the large-scale affinity changes caused by global rearrangement of the lectin domain during the shift between tense and relaxed conformations, we found dynamic binding loop differences within FimH_LD_ that underlie the altered binding affinity of *K. pneumoniae* FimH_LD_. This phenomenon has also been recently reported for *Acinetobacter baumannii* Abp1D and Abp2D CUP fimbrial adhesin receptor binding domains, in which dynamics of an anterior binding loop modulate receptor binding domain affinity (42). Thus, there may be multiple scales of conformational dynamics within two-domain adhesins that interact to achieve allosteric fine-tuning of binding affinity. Further work will explore the prevalence and function of adhesin dynamics in other CUP pili systems.

It is possible that the conformational state of *K. pneumoniae* FimH sacrifices binding affinity to terminal monomannose to allow binding to a wider range of mannosylated glycans. Recombinant expression of *K. pneumoniae* FimH_S62A_, a variant found in a cohort of clinical uropathogenic isolates, increased binding of bacteria to monomannosylated and trimannosylated surfaces independent of shear force stress compared to *K. pneumoniae* WT FimH, which bound well only to trimannosylated surfaces under high shear force (43). The tense-shifted UPEC FimH mutant (A62S) in UTI89 still achieves a low HA titer (32), while *K. pneumoniae* FimH WT fails to produce an HA titer, suggesting that the conformational shift toward a low-affinity binding state may reflect more extensive interdomain interactions in *K. pneumoniae* FimH compared to *E. coli* FimH.

Antibiotic-sparing FimH-targeted therapeutics, such as mannosides and FimH-based vaccine strategies have been shown to successfully clear or prevent UPEC UTI in animal models and early clinical studies (5–7, 36, 44). The present study sets the stage for future investigations to evaluate the effectiveness of these strategies in models and clinical cases of *K. pneumoniae* UTI. Given increasing prevalence, morbidity, and resistance to standard-of-care antibiotics in UTI, particularly those caused by *K. pneumoniae*, such approaches are of considerable importance.

## Methods

### Bioinformatics.

To construct the whole-genome and FimH phylogenetic trees, initial assembly files were downloaded from Institut Pasteur *Klebsiella* MLST database on July 29, 2022. *K. pneumoniae* FimH protein sequences were searched for identical *E. coli* sequences using NCBI protein BLAST on July 3, 2024 (45). *SI Appendix* *c*ontains detailed phylogenetic methods on tree constructions and visualizations.

### Bacterial Strains and Cloning.

Human catheter isolates were obtained as described in (37) and deidentified prior to use in our study. *fimH* sequences were obtained by PCR and Sanger sequencing. Primers, plasmids, and bacterial stains used are listed in *SI Appendix*, Table S3–S5. All bacterial strains were previously published except for the TOP52 *fimS* and *fimH* chromosomal variants. Chromosomal mutations *fimS* and *fimH* in TOP52 were generated as described previously using double allelic exchange with the pKOV vector (8, 46). Briefly, for TOP52 *fimH* mutants, a pKOV-*fimH* plasmid was created and mutations were made using QuickChange mutagenesis (47). Double allelic exchange selection occurred after transformation into TOP52. A *fimS*-LON pKOV vector was created by cloning the *fimS* sequence from TOP52 *fimK* deletion (23) into pKOV to create a mixed population of pKOV-*fimS* with both ON and OFF orientations. The mixed population was then subject to QuickChange mutagenesis (changing left inverted repeat to 5’-gccttagtc-3’ with primers designed to only amplify the ON FimS orientation), and the pKOV-*fimS*-LON plasmid was isolated and used for double allelic exchange. Kan^R^ and Tet^R^ TOP52 strains were created using the red recombinase method as previously described to introduce the kanamycin resistance gene from pKD4 and tetracycline resistance gene from pBR322, separately, into locus D1637_01605 in TOP52 (8, 48, 49).

*K. pneumoniae* gene sequences for *fimH_LD_* (N-terminal domain residues 1 to 160 with 6x his-tagged c terminus), full-length *fimH*, and *fimC_his_* were cloned from *K. pneumoniae* strain TOP52 genomic DNA using standard PCR, restriction enzyme, and ligation cloning techniques. *K. pneumoniae* gene sequences for *fimH_LD_* and *fimC* from TOP52 were cloned into pTRC99a and pBAD33 vectors, respectively. The gene encoding *fimC* from *E. coli* strain J96 was cloned in pTRC99a and *fimG* from *E. coli* UTI89 was cloned into pBAD33. pBAD33 vectors containing UTI89 FimH were described previously (33). All plasmid mutations and additions of c-terminal 6 × histidine tags were made using QuickChange mutagenesis (47). Optimal ribosomal binding sites (*SI Appendix*, Table S3) were cloned into plasmids expressing *K. pneumoniae fimC_his_* and *fimH* to improve expression and match the UTI89 *fimH* and *fimC* plasmids (27).

### Protein Purification.

*K. pneumoniae* FimH_LD-his_, FimC_his_H complexes, and *E. coli* FimH truncate from J96 were purified from periplasm preparations in *E. coli* strain C600 using affinity and ion-exchange chromatography as previously described (19, 50). FimCG_db_H variant complexes were made by mixing FimCG_db_ and FimC_his_H at a 3:1 molar ratio in 20 mM MES (pH 5.6) at 30 °C for 16 h. Pure FimCG_db_H was purified from this reaction using a cation (S) exchange column. Proteins were stored in 20 mM MES pH 5.6.

### Crystallography.

TOP52 FimH_LD_ (7.0 mg/ml) in 20 mM Tris pH 8.0 was crystallized via hanging drop with a 10:1 molar excess of d-mannose in a 1:1 drop with the well solution 0.4 M MgCl_2_, 2.0 M NaCl, and 0.1 M Tris pH 7.0. Crystals formed in 24 to 48 h. Crystals were looped and cryoprotected in 0.2 M MgCl_2_, 2.0 M NaCl, and 0.1 M Tris pH 7.0, and 20% glycerol. Data were collected at ALS 4.2.2 macromolecular crystallography beamline, and the structure was solved by molecular replacement and refinement in Phenix using *E. coli* FimH lectin domain (PDB 1KLF trimmed to the lectin domain) as the search model (*SI Appendix*, Table S6).

### Molecular dynamics simulations.

Molecular dynamics simulations were run with Gromacs 2020.1 (51–53) using the FAST algorithm (54). Detailed *methods* are listed in *SI Appendix*.

### ELISA, BLI, and DSF Protein Binding.

Protein binding measurements by ELISA, BLI, and DSF followed previously standard protocols (27, 42). Detailed *methods* are listed in *SI Appendix*.

### Pilus Purification, Pilus Counts, Hemagglutination Titers, and Western Blotting of Bacteria.

UTI89 LON Δ*fimH* strains complemented with pBAD33-FimH variants were grown in LB with 0.01% arabinose with shaking at 37 °C overnight. TOP52 strains were grown statically 2 × 24 (grown for 24 h and then subcultured 1:1000 for another 24 h) in LB broth at 37 °C, the same conditions as described previously for optimal expression of type 1 pili in *E. coli* (39). Bacteria were normalized by optical density for HA assays and pilus counts by electron microscopy. Pili were isolated by heat extraction followed by salt precipitation (55). Bacterial hemagglutination assays (HAs) were performed with guinea pig erythrocytes (Colorado Serum Company) as previously described (56) with and without the addition of 100 mM methyl α-d-mannopyranoside. *SI Appendix* *f*or detailed *methods*.

### FimS qPCR Phase Assay.

Previously described *fimS* orientation (8) PCR assays were adapted for *K. pneumoniae* TOP52 strains using qPCR. Briefly, a set of three primers were designed based on the TOP52 genome (NCBI JNFE00000000.1) and are listed in *SI Appendix,* Table S3. Genomic DNA was isolated with the Promega Wizard Genomic DNA Kit per the manufacturer’s instructions and quantified using quantitative PCR (qPCR). Amplifications were carried out in a CFX96 Touch Real-Time PCR system (Bio-Rad) per the manufacturer’s instructions. The ΔΔ*C_T_* method was used to calculate the ratio of ON:OFF configurations with *gyrA* gene used as internal reference control.

### 5637 Cell Invasion and Attachment Assays.

5637 (ATCC HTB-9) human bladder epithelial cells were grown and used for attachment and invasion assays as previously specified (27). Detailed *methods* are provided in *SI Appendix*.

### Mouse Infections.

For acute and chronic UTIs, female 6 to 8 wk old C3H/HeN mice (Envigo) were transurethrally inoculated with 50 μL bacterial suspension in PBS (total of ~2 × 10^9^ CFU) according to established mouse models (57, 58). For competitive infection experiments, female 6 to 8 wk old C3H/HeN mice were inoculated with a 50 μL 1:1 mix of bacteria totaling ~2 × 10^9^ CFU. For the murine model of CAUTI, female 6- to 8-wk-old C57BL/6 mice (Charles River) were catheterized with 4 to 5 mm silicon tubing and immediately infected with ~2 × 10^7^ CFU of bacteria as previously described (40). Organ and catheter titers were determined by dilution and plating. All studies were approved and performed in accordance with the guidelines set by the Institutional Animal Care and Use Committee at Washington University School of Medicine under protocol 21-0341 (Animal Welfare Assurance # D16-00245).

## Supplementary Material

Appendix 01 (PDF)

## Data Availability

Structure of biological macromolecule data have been deposited in Protein Data Bank (9BOG) (59). All study data are included in the article and/or *SI Appendix*.

## References

[1] B. Foxman, Urinary tract infection syndromes: Occurrence, recurrence, bacteriology, risk factors, and disease burden. Infect. Dis. Clin. North Am. **28**, 1–13 (2014). 24484571 10.1016/j.idc.2013.09.003

[2] C. W. Seymour , Time to treatment and mortality during mandated emergency care for sepsis. N. Engl. J. Med. **376**, 2235–2244 (2017). 28528569 10.1056/NEJMoa1703058 PMC5538258

[3] A. L. Flores-Mireles, J. N. Walker, M. Caparon, S. J. Hultgren, Urinary tract infections: Epidemiology, mechanisms of infection and treatment options. Nat. Rev. Microbiol. **13**, 269–284 (2015). 25853778 10.1038/nrmicro3432 PMC4457377

[4] V. De Lastours, B. Foxman, Urinary tract infection in diabetes: Epidemiologic considerations topical collection on genitourinary infections. Curr. Infect Dis. Rep. **16**, 1–6 (2014). 10.1007/s11908-013-0389-2 24407547

[5] C. K. Cusumano , Treatment and prevention of urinary tract infection with orally active FimH inhibitors. Sci. Transl. Med. **3**, 109ra115 (2011). 10.1126/scitranslmed.3003021 PMC3694776 22089451

[6] S. Langermann , Prevention of mucosal Escherichia coli infection by FimH-adhesin-based systemic vaccination. Science **1979**, 607–611 (1997). 10.1126/science.276.5312.607 9110982

[7] C. M. Starks , Optimization and qualification of an assay that demonstrates that a FimH vaccine induces functional antibody responses in women with histories of urinary tract infections. Hum. Vaccin. Immunother **17**, 283–292 (2021). 32701396 10.1080/21645515.2020.1770034 PMC7872045

[8] D. A. Rosen , Molecular variations in Klebsiella pneumoniae and Escherichia coli FimH affect function and pathogenesis in the urinary tract. Infect. Immun. **76**, 3346–3356 (2008). 18474655 10.1128/IAI.00340-08 PMC2446687

[9] M. M. Barnhart , PapD-like chaperones provide the missing information for folding of pilin proteins. Proc. Natl. Acad. Sci. U.S.A. **97**, 7709–7714 (2000). 10859353 10.1073/pnas.130183897 PMC16609

[10] D. Choudhury , X-ray structure of the FimC-FimH chaperone-adhesin complex from uropathogenic Escherichia coli. Science **1979**, 1061–1066 (1999). 10.1126/science.285.5430.1061 10446051

[11] F. G. Sauer , Structural basis of chaperone function and pilus biogenesis. Science **1979**, 1058–1061 (1999). 10.1126/science.285.5430.1058 10446050

[12] F. G. Sauer, J. S. Pinkner, G. Waksman, S. J. Hultgren, Chaperone priming of pilus subunits facilitates a topological transition that drives fiber formation. Cell **111**, 543–551 (2002). 12437927 10.1016/s0092-8674(02)01050-4

[13] K. W. Dodson, F. Jacob-Dubuisson, R. T. Striker, S. J. Hultgren, Outer-membrane PapC molecular usher discriminately recognizes periplasmic chaperone-pilus subunit complexes. Proc. Natl. Acad. Sci. U.S.A. **90**, 3670–3674 (1993). 8097321 10.1073/pnas.90.8.3670 PMC46363

[14] M. Nishiyama, T. Ishikawa, H. Rechsteiner, R. Glockshuber, Reconstitution of pilus assembly reveals a bacterial outer membrane catalyst. Science **1979**, 376–379 (2008). 10.1126/science.1154994 18369105

[15] M. Du , Handover mechanism of the growing pilus by the bacterial outer-membrane usher FimD. Nature **562**, 444–447 (2018). 30283140 10.1038/s41586-018-0587-z PMC6309448

[16] H. Remaut , Donor-strand exchange in chaperone-assisted pilus assembly proceeds through a concerted β strand displacement mechanism. Mol. Cell **22**, 831–842 (2006). 16793551 10.1016/j.molcel.2006.05.033 PMC7617774

[17] E. T. Saulino, D. G. Thanassi, J. S. Pinkner, S. J. Hultgren, Ramifications of kinetic partitioning on usher-mediated pilus biogenesis. EMBO J. **17**, 2177–2185 (1998). 9545231 10.1093/emboj/17.8.2177 PMC1170562

[18] I. Le Trong , Structural basis for mechanical force regulation of the adhesin fimh via finger trap-like β sheet twisting. Cell **141**, 645–655 (2010). 20478255 10.1016/j.cell.2010.03.038 PMC2905812

[19] C. S. Hung , Structural basis of tropism of Escherichia coli to the bladder during urinary tract infection. Mol. Microbiol. **44**, 903–915 (2002). 12010488 10.1046/j.1365-2958.2002.02915.x

[20] G. Zhou , Uroplakin Ia is the urothelial receptor for uropathogenic Escherichia coli: Evidence from in vitro FimH binding. J. Cell Sci. **114**, 4095–4103 (2001). 11739641 10.1242/jcs.114.22.4095

[21] L. K. McLellan , A host receptor enables type 1 pilus-mediated pathogenesis of Escherichia coli pyelonephritis. PLoS Pathog **17**, e1009314 (2021). 33513212 10.1371/journal.ppat.1009314 PMC7875428

[22] G. G. Anderson , Intracellular bacterial biofilm-like pods in urinary tract infections. Science **1979**, 105–107 (2003). 10.1126/science.1084550 12843396

[23] D. A. Rosen , Utilization of an intracellular bacterial community pathway in Klebsiella pneumoniae urinary tract infection and the effects of FimK on type 1 pilus expression. Infect Immun. **76**, 3337–3345 (2008). 18411285 10.1128/IAI.00090-08 PMC2446714

[24] D. A. Rosen, T. M. Hooton, W. E. Stamm, P. A. Humphrey, S. J. Hultgren, Detection of intracellular bacterial communities in human urinary tract infection. PLoS Med. **4**, e329 (2007). 18092884 10.1371/journal.pmed.0040329 PMC2140087

[25] M. A. Mulvey, J. D. Schilling, S. J. Hultgren, Establishment of a persistent Escherichia coli reservoir during the acute phase of a bladder infection. Infect Immun. **69**, 4572–4579 (2001). 11402001 10.1128/IAI.69.7.4572-4579.2001 PMC98534

[26] M. A. Mulvey , Induction and evasion of host defenses by type 1-piliated uropathogenic Escherichia coli. Science **1979**, 1494–1497 (1998). 10.1126/science.282.5393.1494 9822381

[27] V. Kalas , Evolutionary fine-tuning of conformational ensembles in FimH during host-pathogen interactions. Sci. Adv. **3**, e1601944 (2017). 28246638 10.1126/sciadv.1601944 PMC5302871

[28] M. M. Sauer , Catch-bond mechanism of the bacterial adhesin FimH. Nat. Commun. **7**, 1–13 (2016). 10.1038/ncomms10738 PMC4786642 26948702

[29] P. Aprikian , Interdomain interaction in the FimH adhesin of Escherichia coli regulates the affinity to mannose. J. Biol. Chem. **282**, 23437–23446 (2007). 17567583 10.1074/jbc.M702037200

[30] P. Magala, R. E. Klevit, W. E. Thomas, E. V. Sokurenko, R. E. Stenkamp, RMSD analysis of structures of the bacterial protein FimH identifies five conformations of its lectin domain. Proteins **88**, 593 (2020). 31622514 10.1002/prot.25840 PMC7058522

[31] W. E. Thomas, E. Trintchina, M. Forero, V. Vogel, E. V. Sokurenko, Bacterial adhesion to target cells enhanced by shear force. Cell **109**, 913–923 (2002). 12110187 10.1016/s0092-8674(02)00796-1

[32] S. L. Chen , Positive selection identifies an in vivo role for FimH during urinary tract infection in addition to mannose binding. Proc. Natl. Acad. Sci. U.S.A. **106**, 22439–22444 (2009). 20018753 10.1073/pnas.0902179106 PMC2794649

[33] D. J. Schwartz , Positively selected FimH residues enhance virulence during urinary tract infection by altering FimH conformation. Proc. Natl. Acad. Sci. U.S.A. **110**, 15530–15537 (2013). 24003161 10.1073/pnas.1315203110 PMC3785778

[34] S. G. Stahlhut , Population variability of the FimH Type 1 fimbrial adhesin in klebsiella pneumoniae. J. Bacteriol. **191**, 1941 (2009). 19151141 10.1128/JB.00601-08 PMC2648365

[35] A. Wellens , The tyrosine gate as a potential entropic lever in the receptor-binding site of the bacterial adhesin FimH. Biochemistry **51**, 4790–4799 (2012). 22657089 10.1021/bi300251r

[36] L. Mydock-McGrane , Antivirulence C-mannosides as antibiotic-sparing, oral therapeutics for urinary tract infections. J. Med. Chem. **59**, 9390–9408 (2016). 27689912 10.1021/acs.jmedchem.6b00948 PMC5087331

[37] T. M. Nye , Microbial co-occurrences on catheters from long-term catheterized patients. Nat. Commun. **15**, 1–13 (2024). 38168042 10.1038/s41467-023-44095-0 PMC10762172

[38] D. I. Kisiela , Toggle switch residues control allosteric transitions in bacterial adhesins by participating in a concerted repacking of the protein core. PLoS Pathog **17**, e1009440 (2021). 33826682 10.1371/journal.ppat.1009440 PMC8064603

[39] S. E. Greene, M. E. Hibbing, J. Janetk, S. L. Chen, S. J. Hultgren, Human urine decreases function and expression of type 1 pili in uropathogenic Escherichia coli. mBio **6**, e00820-15 (2015). 26126855 10.1128/mBio.00820-15 PMC4488945

[40] P. S. Guiton, C. S. Hung, L. E. Hancock, M. G. Caparon, S. J. Hultgren, Enterococcal biofilm formation and virulence in an optimized murine model of foreign body-associated urinary tract infections. Infect Immun. **78**, 4166–4175 (2010). 20696830 10.1128/IAI.00711-10 PMC2950371

[41] C. N. Murphy, M. S. Mortensen, K. A. Krogfelt, S. Clegg, Role of klebsiella pneumoniae type 1 and type 3 fimbriae in colonizing silicone tubes implanted into the bladders of mice as a model of catheter-associated urinary tract infections. Infect Immun. **81**, 3009–3017 (2013). 23753626 10.1128/IAI.00348-13 PMC3719564

[42] K. O. Tamadonfar , Structure–function correlates of fibrinogen binding by Acinetobacter adhesins critical in catheter-associated urinary tract infections. Proc. Natl. Acad. Sci. U.S.A. **120**, e2212694120 (2023). 36652481 10.1073/pnas.2212694120 PMC9942807

[43] S. G. Stahlhut , Comparative structure-function analysis of mannose-specific FimH adhesins from klebsiella pneumoniae and escherichia coli. J. Bacteriol. **191**, 6592–6601 (2009). 19734306 10.1128/JB.00786-09 PMC2795292

[44] C. N. Spaulding , Selective depletion of uropathogenic E. coli from the gut by a FimH antagonist. Nature **546**, 528–532 (2017). 28614296 10.1038/nature22972 PMC5654549

[45] S. F. Altschul , Gapped BLAST and PSI-BLAST: A new generation of protein database search programs. Nucleic Acids Res. **25**, 3389–3402 (1997). 9254694 10.1093/nar/25.17.3389 PMC146917

[46] A. J. Link, D. Phillips, G. M. Church, Methods for generating precise deletions and insertions in the genome of wild-type Escherichia coli: Application to open reading frame characterization. J. Bacteriol. **179**, 6228–6237 (1997). 9335267 10.1128/jb.179.20.6228-6237.1997 PMC179534

[47] H. Liu, J. H. Naismith, An efficient one-step site-directed deletion, insertion, single and multiple-site plasmid mutagenesis protocol. BMC Biotechnol. **8**, 91 (2008). 19055817 10.1186/1472-6750-8-91 PMC2629768

[48] F. Bolivar , Construction and characterization of new cloning vehicle. II. A multipurpose cloning system. Gene **2**, 95–113 (1977). 344137

[49] K. A. Datsenko, B. L. Wanner, One-step inactivation of chromosomal genes in Escherichia coli K-12 using PCR products. Proc. Natl. Acad. Sci. U.S.A. **97**, 6640–6645 (2000). 10829079 10.1073/pnas.120163297 PMC18686

[50] L. N. Slonim, J. S. Pinkner, C. I. Brändén, S. J. Hultgren, Interactive surface in the PapD chaperone cleft is conserved in pilus chaperone superfamily and essential in subunit recognition and assembly. EMBO J. **11**, 4747 (1992). 1361168 10.1002/j.1460-2075.1992.tb05580.x PMC556950

[51] M. J. Abraham , GROMACS: High performance molecular simulations through multi-level parallelism from laptops to supercomputers. SoftwareX **1–2**, 19–25 (2015).

[52] K. Lindorff-Larsen , Improved side-chain torsion potentials for the Amber ff99SB protein force field. Proteins: Struct., Funct., Bioinf. **78**, 1950–1958 (2010). 10.1002/prot.22711 PMC2970904 20408171

[53] W. L. Jorgensen, J. Chandrasekhar, J. D. Madura, R. W. Impey, M. L. Klein, Comparison of simple potential functions for simulating liquid water. J. Chem. Phys. **79**, 926–935 (1983).

[54] M. I. Zimmerman, G. R. Bowman, FAST conformational searches by balancing exploration/exploitation trade-offs. J. Chem. Theory Comput. **11**, 5747–5757 (2015). 26588361 10.1021/acs.jctc.5b00737

[55] M. J. Kuehn, J. Heuser, S. Normark, S. J. Hultgren, P pili in uropathogenic E. coli are composite fibres with distinct fibrillar adhesive tips. Nature **356**, 252–255 (1992). 1348107 10.1038/356252a0

[56] S. J. Hultgren, W. R. Schwan, A. J. Schaeffer, J. L. Duncan, Regulation of production of type 1 pili among urinary tract isolates of Escherichia coli. Infect Immun. **54**, 613 (1986). 2877947 10.1128/iai.54.3.613-620.1986 PMC260213

[57] T. J. Hannan, I. U. Mysorekar, C. S. Hung, M. L. Isaacson-Schmid, S. J. Hultgren, Early Severe Inflammatory Responses to Uropathogenic E. coli Predispose to Chronic and Recurrent Urinary Tract Infection. PLoS Pathog. **6**, e1001042 (2010). 20811584 10.1371/journal.ppat.1001042 PMC2930321

[58] T. J. Hannan, D. A. Hunstad, A murine model for escherichia coli urinary tract infection. Methods in Mol. Biol. **1333**, 159–175 (2016). 26468108 10.1007/978-1-4939-2854-5_14 PMC4624421

[59] R. M. Bitter, M. Zimmerman, S. Hultgren, P. Yuan, Structural basis for adhesin secretion by the outer-membrane usher in type 1 pili. Protein Data Bank (PDB). 10.2210/pdb9BOG/pdb. Deposited 2 May 2024. PMC11459180 39316053

