# Supplementary material for: Conformational ensembles in Klebsiella pneumoniae FimH impact uropathogenesis
Source: Proc Natl Acad Sci U S A. 2024 Sep 17;121(39):e2409655121. doi: 10.1073/pnas.2409655121 (PMC11441496; doi:10.1073/pnas.2409655121)
Supplement: Supplementary file 1 — Appendix 01 (PDF) [file pnas.2409655121.sapp.pdf]

PNAS

Supporting Information for

Conformational Ensembles in *Klebsiella pneumoniae* FimH Impact Uropathogenesis

Edward D. B. Lopatto[1,2], Jerome S. Pinkner[1,2], Denise A. Sanick[1,2], Robert F. Potter[3], Lily X. Liu[1,2], Jesús Bazán Villicaña[1,2], Kevin O. Tamadonfar[1,2], Yijun Ye[1,2], Maxwell I. Zimmerman[1,2], Nathaniel C. Gualberto[1,2], Karen W. Dodson[1,2], James W. Janetka[1,4], David A. Hunstad[1,2,3], and Scott J. Hultgren[1,2]

[1] Department of Molecular Microbiology, Washington University School of Medicine, St. Louis, MO 63110
[2] Center for Women's Infectious Disease Research, Washington University School of Medicine, St. Louis, MO 63110
[3] Department of Pediatrics, Washington University School of Medicine, St. Louis, MO 63110
[4] Department of Biochemistry and Molecular Biophysics, Washington University School of Medicine, St. Louis, MO 63110

Scott J. Hultgren
Email: hultgren@wustl.edu

This PDF file includes:

Detailed Materials and Methods
Figures S1 to S3
Tables S1 to S6
SI References

Detailed Materials and Methods

Bioinformatics

To construct the whole-genome phylogenetic tree, initially assembly files were downloaded from Institut Pasteur Klebsiella MLST database on July 29, 2022. Files were initially curated for quality and diverse representation of depositor source. All fasta files were initially annotated for open reading frames using prokka v1.14.6 (prokka ${ID}.fas --outdir ${ID} --locustag ${ID} --mincontiglen 500 --prefix ${ID} --force --notrna --norrna --centre X --compliant)(1). Panaroo v1.0 was used to construct the pan-genome and core-genome alignment (panaroo -i ${indir}/*.gff -o ${outdir} --clean-mode moderate -a core --core_threshold .99 -t 12 --search_radius 10 --refind_prop_match 100 -t ${SLURM_CPUS_PER_TASK})(2). Single nucleotide polymorphisms from the core-genome alignment were identified with SNP-sites v2.5.1 (snp-sites -mvp -o output_file_name core_gene_alignment.aln) and the resulting alignment file was used to create an approximate-maximum likelihood phylogenetic tree with FastTree v2.1.9 (FastTree -nt -gtr -gamma snp_sites.snp_sites.aln > fasttree.nwk)(3, 4). The newick format was viewed in the Interactive Tree of Life (iTOL v6) webserver (https://itol.embl.de/) (5). MLST identification (mlst --scheme klebsiella ${ID}.fas) was performed on the assembly files using PubMLST v2.23.0 (https://github.com/tseemann/mlst). FimH sequence were extracted from the prokka genome annotation and aligned using MUSCLE (muscle -in fimH.fasta -out fimH.aln) and converted into an approximate-maximum likelihood phylogenetic tree with FastTree v2.1.9 (FastTree -gtr -gamma fimH.aln > fimH.nwk) and viewed using the iTOL v6 webserver.

Molecular dynamics simulations

Molecular dynamics simulations were run with Gromacs 2020.1 at 310 K using the AMBER99SB-ILDN force field with explicit TIP3P solvent (6–8). Simulations for UPEC $FimH_{LD}$ and K. pneumoniae $FimH_{LD}$ were prepared by placing the starting structures (PDB IDs: 1KLF trimmed to lectin domain and 9AT9, respectively) without ligands in a dodecahedron box that extends 1.0 Å beyond the protein in any dimension. Each system was then solvated and energy minimized with a steepest descents algorithm until the maximum force fell below 100 kJ/mol/nm using a step size of 0.01 nm and a cutoff distance of 1.2 nm for the neighbor list, Coulomb interactions, and van der Waals interactions. For production runs, all bonds were constrained with the LINCS algorithm and virtual sites were used to allow a 4 fs time step (9, 10). Cutoffs of 1.0 nm were used for the neighbor list, Coulomb interactions, and van der Waals interactions. The Verlet cutoff scheme was used for the neighbor list. The stochastic velocity rescaling (v-rescale) (11) thermostat was used to hold the temperature at 310 K. Conformations were stored every 20 ps.

The FAST algorithm (12) was used to enhance conformational sampling of conformations distant from the starting state. For each system, FAST-RMSD simulations were run for 6 rounds with 10 simulations per round, where each simulation was 50 ns in length (3 μs aggregate simulation per system). The FAST-RMSD ranking function favored restarting simulations from states that maximized the RMSD of backbone heavy-atoms to the starting conformation. Additionally, a similarity penalty was added to the ranking to promote conformational diversity in starting structures, as has been described previously (13).
Markov State models (MSMs) were built from the FAST simulation data using enspara (14). An MSM is a network representation of a free-energy landscape, where nodes are discrete conformational states and directed edges are conditional transition probabilities. The state space was defined using backbone heavy atoms (atoms C, Cα, Cβ, N, O), which was clustered with a k-centers algorithm based on RMSD between conformations until every cluster center had a radius less than 1.0 Å. Following clustering, an MSM was built by row-normalizing the observed transition counts, at a lag-time of 4 ns, with a small pseudo-count as a prior (15).

ELISA

For assessment of FimH binding to mannosylated proteins, ELISAs were performed using Microlon600 (Greiner) plates coated overnight in bovine submaxillary mucin (10 ug/mL). The plate was then blocked for 2 h with 1x PBS + 1% BSA (blocking buffer). Protein in 1x PBS buffer was added and incubated at 37°C for 1 h. After three washes of 1x PBS + 0.05% tween-20 (wash buffer), FimCH binding was detected by FimH lectin domain anti-sera (rabbit anti-t3,1:5000 dilution) and FimCGH binding was detected by

FimC anti-sera (rabbit, 1:5000 dilution). Primary antibodies were incubated for 1h in blocking buffer. Separate primary antibodies were used as FimH lectin domain anti-sera does not react equally with $FimCG_{bd}H$ variants due to the conformational equilibrium shifts and anti-FimC reacts differently to *E. coli* and *K. pneumoniae* FimC present in $FimC_{his}H$ variants (all $FimCG_{bd}H$ variants have *E. coli* FimC). Plates were then washed three times with wash buffer. Anti-rabbit-HRP secondary (KPL; 1:5000) was added for 1h in blocking buffer, washed three times again with wash buffer, and developed with tetramethylbenzidine substrate (BD Biosciences) and quenched with 1M H_2SO_4. Absorbance was measured at 450 nm. To quantify binding area under the curve (AUC), quantified in PRISM 10 (GraphPad), was chosen due to the lack of saturation in the $FimCG_{DB}H$ binding curves.

BLI

An Octet Red 96-well unit with streptavidin pins was used for BLI measurements. Pins were coated in biotinylated BSA-mannose (10ug/ml). Biotinylated BSA-mannose was made by biotinylating BSA-mannose (Dextra) using EZ NH4-PEG4-biotinylation reagent (ThermoScientific). Pins were blocked in 1x PBS + 1% BSA + 0.05% tween-20 then $FimH_{LD}$ at a series of concentrations was allowed to bind for 600 seconds followed by dissociation in blocking buffer for another 600 seconds. Data and analysis were collected and performed on the Octet Data Acquisition 9 software.

Differential scanning fluorimetry

DSF of $FimH_{LD}$ with mannoside compounds was performed as described previously (16). Briefly, $FimH_{LD}$ (1uM final concentration) was mixed with ligands (10uM final concentration) and SYPRO Orange (Sigma-Aldrich) in a 50uL reaction volume buffered by 1x PBS. The reaction was measured using the “HEX” fluorescence channel in a CFX96 thermocycler (Bio-Rad) in a reading cycle that spans 20° to 90°C in 0.5°C increments of 15 s. Mannosides are described in SI Appendix, Table S1, and synthesis and other properties are described in prior reports (17, 18).

Pilus expression and purifications

UTI89 LON Δ*fimH* strains complemented with pBAD33-FimH variants were grown in LB+0.01% arabinose shaking 37°C overnight for pilus purifications, HA assays, pilus counts. TOP52 1721, a *K. pneumoniae* cystitis isolate, (referred to as TOP52) strains were grown statically 2x24 in LB 37°C, the same conditions as described previously for optimal expression of type 1 pili in *E. coli* (19). Pili were isolated by washing cells in 1.0 mM Tris pH 8.0 and heated to 65°C in a water bath for 1 h. Cells were pelleted and supernatants containing pili were recovered. Pili were salt precipitated from solution with 300 mM NaCl and 100 mM $MgCl_2$. Precipitated pili were harvested by centrifugation and resuspended in 1.0 mM Tris pH 8.0 and an additional centrifugation was used to remove contaminants.

Hemagglutination titers

Bacterial hemagglutination assays (HAs) were performed with guinea pig erythrocytes (Colorado Serum Company) as previously described (20) with and without the addition of 100 mM methyl α-D-mannopyranoside. HA with purified pili was performed similarly with the following modifications. Purified pili were normalized by absorbance at 280nM equal to 0.50 and confirmed normalized amount of FimA by Coomassie stain. Pili were titrated 2-fold in 1x PBS + type-1 pili antisera (1:25 final concentration) and then red blood cells were added to each well.

Pilus counts

Bacterial statically grown 2x24 were subject to electron microscopy to count pili as previously described (21). 200 representative individual bacteria were assessed for each strain indicated and binned into a category of either bald, low-piliation, medium-piliation, or high-piliation.

Western blotting

For bacterial preparations, bacteria grown 2x24 statically was normalized to optical density at 600nm of 1. Purified pili were normalized by absorbance at 280 nm. Both bacterial and pili preparations were acid-treated for SDS-PAGE and immunoblotting as described previously (22). To measure FimA, rabbit anti-type 1 pili (1:2000) was used. Mouse anti-GAPDH (1:10,000, Thermo Fisher) was used to ensure equal loading of bacterial preps. To measure FimH, rabbit anti-T3 (1:5000) was used. Secondary antibodies of

goat anti-rabbit-HRP (1:10,000, KPL) and sheep anti-mouse-HRP (1:5000, GE) was used to detect followed by development with SuperSignal^{TM} West Femto Maximum Sensitivity Substrate (Thermo Fisher). Images were obtained on a BioRad ChemiDoc system.

5637-cell Invasion and attachment assays
5637 (ATCC HTB-9) human bladder epithelial cells were grown and used for invasion and attachment assays as previously specified (16). Briefly, bacteria were normalized to an optical density 600nM of 0.5 in LB and added to well of confluent cells at a multiplicity of infection of 10. Bacteria were allowed to incubate with cells for 2 hours at 37°C and then wells were washed 5 times with 1x PBS. Wells were then either: i) treated with Triton X-100 for 45 min to lyse host cells and supernatants plated to determine levels of adherent bacteria; or ii) treated with 100 ug/ml gentamicin (Sigma-Aldrich) for 2 hours to kill extracellular bacteria followed by washing 3 times and lysing with Triton X-100 to determine intracellular bacteria. Lysates from each condition was titer plated on LB and grown overnight 37°C to measure bacterial CFUs.

Figures
Figures 2-5 were created or assembled, at least in part, in Biorender (Biorender.com).

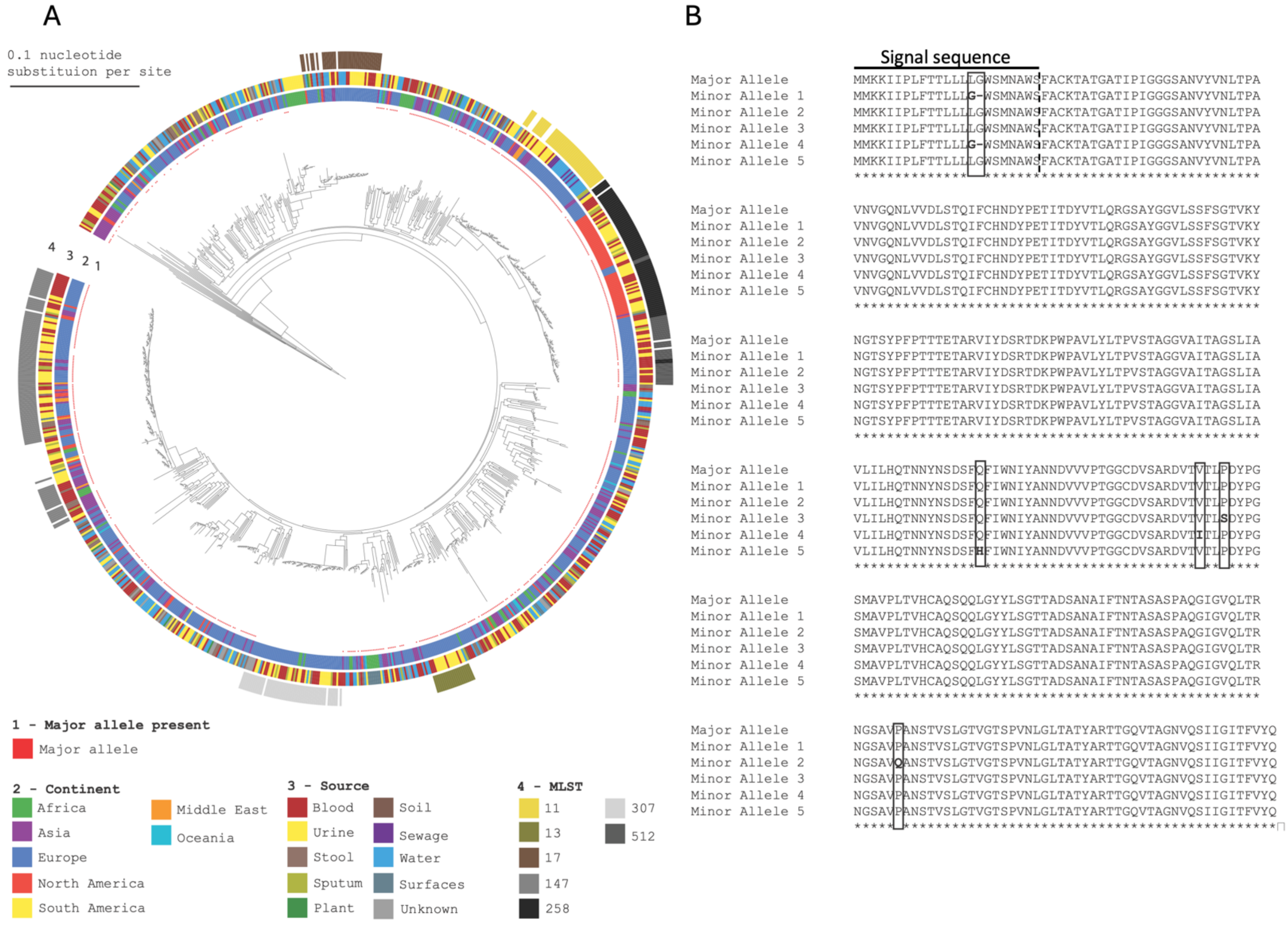

Figure S1. A) Whole-genome phylogeny of *K. pneumoniae* strains used in FimH phylogeny (n=977). Inner most ring red dots indicate major FimH allele present. Second most inner ring represents geographic region of isolation. Third most inner ring represent source of strain. Outer ring denotes MLST. B) Five most prevalent minor FimH amino acid alleles.

Table S1. Mannoside compounds used in DSF binding studies

Name	Structure
5Z240	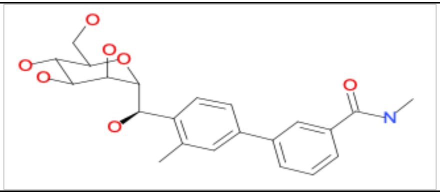
Fim1033	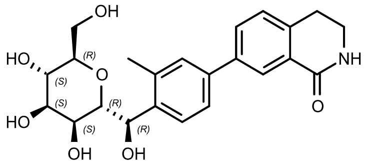
Fim0128	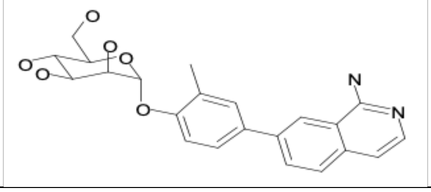
5Z254	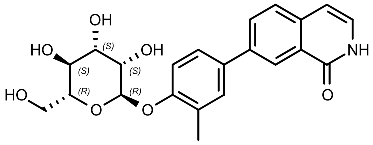
Fim2065	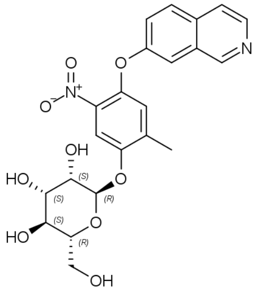
Fim1067	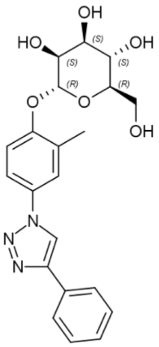

CJ75	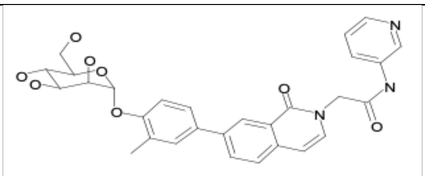
3ZFH265	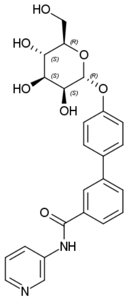
2ZFH265	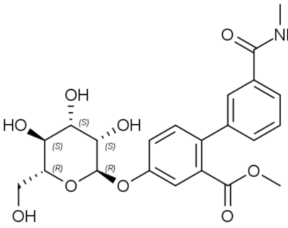
3ZFH280	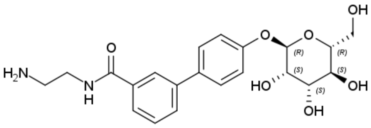
3ZFH18	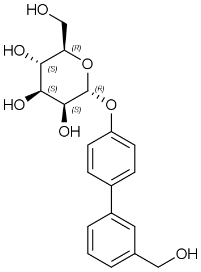

Fim1006	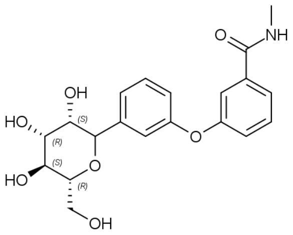

Table S2. Description of human CAUTI *K. pneumoniae* Isolates

Patient	Time (days) between first and last sample	Isolate (Patient # – Sample #)		FimH variant from TOP52	Antibiotics used during infection
82	446	First	82-02	TOP52	Methenamine<br>Duricef<br>Nitrofurantoin
		Last	82-11	TOP52	
84	271	First	84-02	I94V/A105T/G244S	Keflex
		Last	84-06	I94V/A105T/G244S	
85	410	First	85-00	TOP52	None
		Last	85-10	TOP52	
89	59	First	89-01	T74P	None
		Last	89-03	T74P	
99	183	First	99-02	TOP52	Cefazolin<br>Keflex
		Last	99-08	TOP52	
100	637	First	100-05	V36I/G66S	Bactrim<br>Macrobid<br>Ciprofloxacin
		Last	100-11	V36I/G66S	
112	184	First	112-01	TOP52	Ciprofloxacin
		Last	112-03	TOP52	
121	253	First	121-00	TOP52	Azithromycin<br>Ceftriaxone
		Last	121-08	TOP52	
123	103	First	123-06	TOP52	Bactrim<br>Amoxicillin<br>Clavulanic<br>Nitrofurantoin
		Last	123-09	TOP52	
134	350	First	134-00	TOP52	Cephalexin<br>Cefazolin<br>Bactrim
		Last	134-11	TOP52	

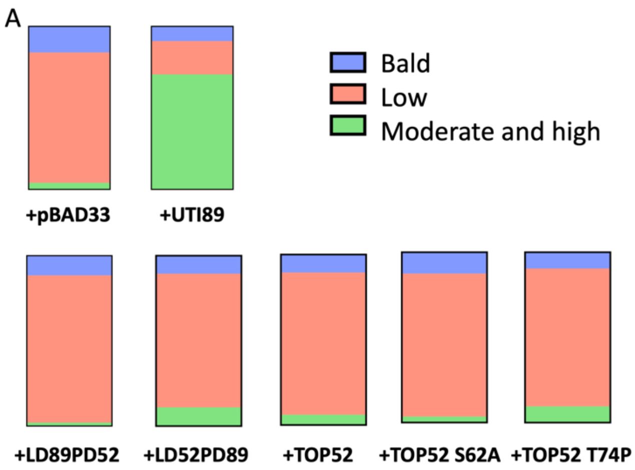

Figure S2. Piliation of UTI89 LON Δ*fimH* strains. A) Pilus counts and B) representative FimA and FimH blots of select strains used in complementing UTI89 LON Δ*fimH*. Labels indicate which FimH plasmid variant or empty vector was used to complement. For pilus counts in (A), 200 bacteria were counted and categorized.

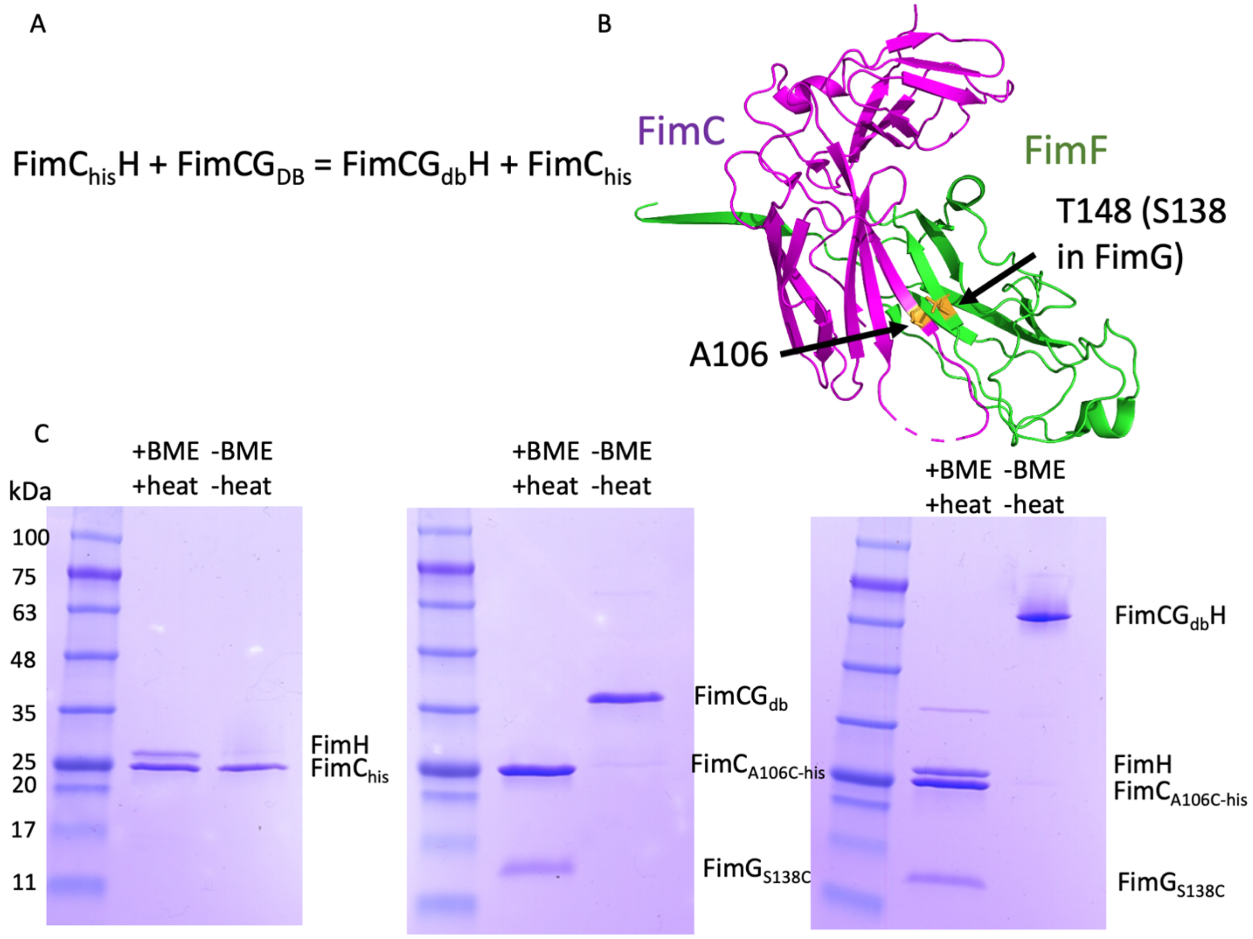

Figure S3. *In vitro* donor strand exchange (DSE) to create $FimCG_{db}H$. A) Overview of DSE reaction. B) Highlight of residues chosen for disulfide bond in FimCG (using structure of FimCF from PDB 4J3O as no FimCG structure currently exists). Coomasie gel of C) $FimC_{his}H$), D) $FimCG_{db}$, and E) $FimCG_{db}H$. (D) and (E) are shown with and without BME and heat to show complex formation.

Table S3. Primers used for cloning, diagnostics, and qPCR assays

Name	Description	Sequence (5'-3')
TOP52 $FimH_{LD}$ F	$FimH_{LD}$ his into ptrc99A	ggcggaattccgcaaggcaccattcagg
TOP52 $FimH_{LD}$ R	$FimH_{LD}$ his into ptrc99A	ggcccagatctattaatggtgatggtgatgatggccgccggtggggacc
TOP52 FimH F	FimH into pBAD33	taagcaggtaccatgaaaaaaataatcccctg
TOP52 FimH R	FimH into pBAD33	tgcttaaagctttcattgatagacaaaggtgat
FimHoptRBS F	FimH optimized RBS	gtttaactttaagaaggagatatacatatgattgtaatgaaaaaaataatcccc
FimHoptRBS R	FimH optimized RBS	ctccttcttaaagttaaacaaaattatttcgagctcgaatcgctagccc
TOP52 $FimC_{his}$ F	$FimC_{his}$ into ptrc	caccatcaccatcaccattaacccagaagcgccccgccgcgcgggacacgctg
TOP52 $FimC_{his}$ R	$FimC_{his}$ into ptrc	tgcttaggatccttacatcaccatcaccatcacgcgcagcacgccattcattttcggcgtcagc
FimCoptRBS F	FimC optimized RBS	ggtacctactagaattcaagaaggagatatacatgtgagcagacaaggagataatgtgag
FimCoptRBS R	FimC optimized RBS	gtatatctccttcttgaattctagtaggtaccgagctcgaattccatggtctgtttcctg
TOP52 FimH F V35E	Quickchange variant	gcaaaacctggaggtcgacctc
TOP52 FimH R V35E	Quickchange variant	aggtcgacctccaggttttgc
TOP52 FimH F D54N	Quickchange variant	atcaccaattacgtgaccctgcagc
TOP52 FimH R D54N	Quickchange variant	tcacgtaataggtgatcgtttccgg
TOP52 FimH F S62A	Quickchange variant	cagcgcggagccgcctatggcggtg
TOP52 FimH R S62A	Quickchange variant	ataggcggctccgcgctgcagggtc
TOP52 FimH F T74P	Quickchange variant	agtttttcaggccccgtgaaatataacgg
TOP52 FimH R T74P	Quickchange variant	atatttcacggggcctgaaaaactcg
TOP52 FimH F I94V	Quickchange variant	accgcgcgggtggtttatgattc

TOP52 FimH R I94V	Quickchange variant	aatcataaaccacccgcgcggtttcc
TOP52 FimH F D96N	Quickchange variant	ggtgatttataattcacggaccgataaaccctg
TOP52 FimH R D96N	Quickchange variant	tcggtccgtgaattataaatcacccgc
TOP52 FimH F A105V	Quickchange variant	accctggccggtcgtcctgtatctg
TOP52 FimH R A105V	Quickchange variant	agatacaggacgaccggccagggtttatc
TOP52 FimH F T114S	Quickchange variant	acgccggtgagctctgccggtgg
TOP52 FimH R T114S	Quickchange variant	actccaccggcagagctcaccggcg
TOP52 FimH F T121K	Quickchange variant	agtggccatcaaagcaggatcg
TOP52 FimH R T121K	Quickchange variant	taacgatcctgctttgatggccactc
TOP52 FimH F H132R	Quickchange variant	ctgatcctgcgtcagaccaacaactac
TOP52 FimH R H132R	Quickchange variant	tgttggtctgacgcaggatcagcacc
TOP52 FimH F Q133K	Quickchange variant	gatcctgcataagaccaacaactac
TOP52 FimH R Q133K	Quickchange variant	ctattgtagttgttggtcttatgcagg
TOP52 FimH F S141D	Quickchange variant	acaatagcgacgacttccagttcatctgg
TOP52 FimH R S141D	Quickchange variant	tgaactggaagtcgtcgctattgtagttg
TOP52 FimH F V163A	Quickchange variant	gctgcgacgcctccgcccgcgatgtc
TOP52 FimH R V163A	Quickchange variant	cgggcggaggcgtcgcagccgc
TOP52 FimH F Q189K	Quickchange variant	tccactgcgcgaaaagccagcagc
TOP52 FimH R Q189K	Quickchange variant	tgctggcttttcgcgcagtggacg

TOP52 FimH F P233Q	Quickchange variant	cagcgccgtcaaggcgaacagcacg
TOP52 FimH R P233Q	Quickchange variant	cgtgctgttcgccttgacggcgctg
TOP52 FimH F G244S	Quickchange variant	tgggcaccgtctccacctcgccg
TOP52 FimH R G244S	Quickchange variant	accggcgaggtggagacggtgcc
Ptrc 5’	Creation of J96 $FimC_{his}$ in ptrc99A	gacaattaatcatccggctc
Ptrc 3’	Creation of J96 $FimC_{his}$ in ptrc99A	ctgttttatcagaccgcttc
J96 FimC his F	Creation of J96 $FimC_{his}$ in ptrc99A	catcatcaccatcaccactaaggatcctctagagtcga
J96 FimC his R	Creation of J96 $FimC_{his}$ in ptrc99A	gtggtgatggtgatgatgttccattacgcccgtcattttgggg
UTI89 FimG F	Cloning FimG into pBAD33	atccggggtacccttgaatatcagtaactggagatgctcatg
UTI89 FimG R	Cloning FimG into pBAD33	atccggaagcttttacaatcatctctttgggttcagctgtagg
E. coli FimC A106C F	Quickchange variant	acgctacagctctgcattatcagccgc
E. coli FimC A106C R	Quickchange variant	ggctgataatgcagagctgtagcgtattctcag
UTI89 FimG S138C F	Quickchange variant	ttcaggcagtgatttgcatcacctatacctac
UTI89 FimG S138C R	Quickchange variant	aggtataggtgatgcaaatcactgcctgaatgg
TOP52 FimHK region F	For creation of pKOV-TOP52 FimHK	ggattttactgtcgatttacaggataacgccgcgaagcag
TOP52 FimHK region R	For creation of pKOV-TOP52 FimHK	gtttgccaaacatgaattcgataacacccgcgaatacgacga
Kpn FimH F	For PCR amplification of *K. pneumoniae* FimH	ccagtcggtacaggtggatgaggccagcc

Kpn FimH R	For PCR amplification of *K. pneumoniae* FimH	gcggctcagaatcaacatcggtaacggcgg
TOP52 FimS F	For creation of pKOV-TOP52 FimS	atccgggcggccgcctttcccgccaacagttttatcaga
TOP52 FimS R	For creation of pKOV-TOP52 FimS	atccggggatccactgataggtcaatgagaaccaggc
TOP52 FimS LON F	Quickchange variant	tcaagccttagtcttttgactcgttgggataaatgatctg
TOP52 FimS LON R	Quickchange variant	aaagactaaggcttgacctgttcgttattttttaattc
F KanR for loci D1637_01605	Creation of TOP52 KanR, pKD4 template	ctgactgagcgcaaggctgaatttgtcgcgttatttccggacgctgccgcaagcactcag
R KanR for loci D1637_01605	Creation of TOP52 KanR, pKD4 template	ctaacaccatgtcagccatgggcttcatcagctcaacgcgagaactccagcatgagatcc
F TetR for loci D1637_01605	Creation of TOP52 tetR, pBR322 template	ctgactgagcgcaaggctgaatttgtcgcgttatttccggtcatgtttgacagcttatca
R TetR for loci D1637_01605	Creation of TOP52 tetR, pBR322 template	ctaacaccatgtcagccatgggcttcatcagctcaacgcgtgttgctcaggtcgcagacg
JBV17	FimS F	agtaatgcggcacgtttcgctg
JBV18	FimS R	gaaataacgaaccggattggcagcc
JBV19	FimS invertible primer	agatcatttatcccaacgagtcaaaatggc
JBV26	GyrA F	ctgctccagcagctctttat
JBV27	GyrA R	gtgcgcgacggtaaatacta

Table S4. Parent plasmids used in this study.

Plasmid	Reference
1. ptrc99a TOP52 $FimH_{LD}$	This study
2. ptrc99a TOP52 $FimC_{his}$	This study
3. pBAD33 TOP52 FimH	This study
4. pBAD33 UTI89 FimH	(16)
5. pBAD33 FimH LD52PD89	(21)
6. pBAD33 FimH LD89PD52	(21)
7. pKOV TOP52 FimS-LON	This study
8. pKOV TOP52 FimHK	This study

Table S5. Parent bacterial strains used in this study.

Bacteria	Reference
1. TOP52 1721	(23)
2. UTI89 LON Δ*fimH*	(19)
3. C600	(16)
4. TOP52 FimS LON	This study

Table S6. Crystal structure refinement statistics.

	Klebsiella pneumoniae FimH Lectin Domain bound to D-Mannose
Wavelength	
Resolution range	35.19 - 1.34 (1.388 - 1.34)
Space group	P 21 21 21
Unit cell	37.539 40.626 101.043 90 90 90
Total reflections	245843 (21635)
Unique reflections	35515 (3188)
Multiplicity	6.9 (6.2)
Completeness (%)	98.92 (91.26)
Mean I/sigma(I)	13.50 (0.63)
Wilson B-factor	14.77
R-merge	0.1014 (2.746)
R-meas	0.1096 (2.999)
R-pim	0.04128 (1.19)
CC1/2	0.999 (0.369)
CC*	1 (0.734)
Reflections used in refinement	35160 (3184)
Reflections used for R-free	1728 (184)
R-work	0.2147 (0.3611)
R-free	0.2318 (0.3627)
CC(work)	0.952 (0.607)
CC(free)	0.956 (0.730)
Number of non-hydrogen atoms	1308
macromolecules	1210
ligands	23
solvent	86
Protein residues	163
RMS(bonds)	0.009
RMS(angles)	1.04
Ramachandran favored (%)	96.27
Ramachandran allowed (%)	3.73
Ramachandran outliers (%)	0
Rotamer outliers (%)	0
Clashscore	2.94
Average B-factor	27.08
macromolecules	26.75
ligands	19.07

solvent	32.77
Number of TLS groups	1

References

1. T. Seemann, Prokka: rapid prokaryotic genome annotation. *Bioinformatics* **30**, 2068–2069 (2014).
2. G. Tonkin-Hill, *et al.*, Producing polished prokaryotic pangenomes with the Panaroo pipeline. *Genome Biol* **21**, 1–21 (2020).
3. A. J. Page, *et al.*, SNP-sites: rapid efficient extraction of SNPs from multi-FASTA alignments. *Microb Genom* **2**, e000056 (2016).
4. M. N. Price, P. S. Dehal, A. P. Arkin, FastTree 2 – Approximately Maximum-Likelihood Trees for Large Alignments. *PLoS One* **5**, e9490 (2010).
5. I. Letunic, P. Bork, Interactive Tree Of Life (iTOL) v5: an online tool for phylogenetic tree display and annotation. *Nucleic Acids Res* **49**, W293–W296 (2021).
6. M. J. Abraham, *et al.*, GROMACS: High performance molecular simulations through multi-level parallelism from laptops to supercomputers. *SoftwareX* **1–2**, 19–25 (2015).
7. K. Lindorff-Larsen, *et al.*, Improved side-chain torsion potentials for the Amber ff99SB protein force field. *Proteins: Structure, Function, and Bioinformatics* **78**, 1950–1958 (2010).
8. W. L. Jorgensen, J. Chandrasekhar, J. D. Madura, R. W. Impey, M. L. Klein, Comparison of simple potential functions for simulating liquid water. *J Chem Phys* **79**, 926–935 (1983).
9. B. Hess, P-LINCS: A Parallel Linear Constraint Solver for Molecular Simulation. *J Chem Theory Comput* **4**, 116–122 (2007).
10. A. K. Mazur, Common molecular dynamics algorithms revisited: Accuracy and optimal time steps of Störmer-Leapfrog integrators. *J Comput Phys* **136**, 354–365 (1997).
11. G. Bussi, D. Donadio, M. Parrinello, Canonical sampling through velocity rescaling. *Journal of Chemical Physics* **126** (2007).
12. M. I. Zimmerman, G. R. Bowman, FAST Conformational Searches by Balancing Exploration/Exploitation Trade-Offs. *J Chem Theory Comput* **11**, 5747–5757 (2015).
13. M. I. Zimmerman, *et al.*, Prediction of New Stabilizing Mutations Based on Mechanistic Insights from Markov State Models. *ACS Cent Sci* **3**, 1311–1321 (2017).
14. J. R. Porter, M. I. Zimmerman, G. R. Bowman, Enspara: Modeling molecular ensembles with scalable data structures and parallel computing. *Journal of Chemical Physics* **150**, 44108 (2019).
15. M. I. Zimmerman, J. R. Porter, X. Sun, R. R. Silva, G. R. Bowman, Choice of Adaptive Sampling Strategy Impacts State Discovery, Transition Probabilities, and the Apparent Mechanism of Conformational Changes. *J Chem Theory Comput* **14**, 5459–5475 (2018).
16. V. Kalas, *et al.*, Evolutionary fine-tuning of conformational ensembles in FimH during host-pathogen interactions. *Sci Adv* **3** (2017).
17. L. Mydock-McGrane, *et al.*, Antivirulence C-Mannosides as Antibiotic-Sparing, Oral Therapeutics for Urinary Tract Infections. *J Med Chem* **59**, 9390–9408 (2016).
18. Z. Han, *et al.*, Structure-based drug design and optimization of mannoside bacterial fimH antagonists. *J Med Chem* **53**, 4779–4792 (2010).
19. S. E. Greene, M. E. Hibbing, J. Janetk, S. L. Chen, S. J. Hultgren, Human urine decreases function and expression of type 1 pili in uropathogenic Escherichia coli. *mBio* **6** (2015).
20. S. J. Hultgren, W. R. Schwan, A. J. Schaeffer, J. L. Duncan, Regulation of production of type 1 pili among urinary tract isolates of Escherichia coli. *Infect Immun* **54**, 613 (1986).
21. D. A. Rosen, *et al.*, Molecular variations in Klebsiella pneumoniae and Escherichia coli FimH affect function and pathogenesis in the urinary tract. *Infect Immun* **76**, 3346–3356 (2008).
22. D. A. Rosen, *et al.*, Utilization of an intracellular bacterial community pathway in Klebsiella pneumoniae urinary tract infection and the effects of FimK on type 1 pilus expression. *Infect Immun* **76**, 3337–3345 (2008).
23. D. A. Rosen, T. M. Hooton, W. E. Stamm, P. A. Humphrey, S. J. Hultgren, Detection of Intracellular Bacterial Communities in Human Urinary Tract Infection. *PLoS Med* **4**, e329 (2007).
